# ‘Priming’ protects *Piper nigrum* L. from *Phytophthora capsici* through reinforcement of phenylpropanoid pathway and possible enhancement of Piperine biosynthesis

**DOI:** 10.3389/fpls.2022.1072394

**Published:** 2022-12-06

**Authors:** M. Indu, B. Meera, KC. Sivakumar, Chidambareswaren Mahadevan, K Mohammed Shafi, B. Nagarathnam, Ramanathan Sowdhamini, Manjula Sakuntala

**Affiliations:** ^1^Plant Disease Biology Laboratory, Rajiv Gandhi Centre for Biotechnology, Trivandrum, India; ^2^Plant Chemetics Laboratory, Department of Biology, University of Oxford, Oxford, United Kingdom; ^3^National Centre for Biological Sciences, Tata Institute of Fundamental Research, Bangalore, India

**Keywords:** Priming, *Piper nigrum*, *Phytophthora capsici*, Glycol chitosan, Piperine, Phenylpropanoid

## Abstract

*Piper nigrum L*. (black pepper), a woody perennial spice crop indigenous to India is positioned at the phylogenetically unique basal lineage of angiosperms. Cultivation of this major spice crop is constrained by rampant fungal and viral infections leading to a lack of disease-free planting material. The major disease that poses severe threat to *P. nigrum* plantations and nurseries is ‘quick wilt’ caused by the oomycete *Phytophthora capsici*, which affects the leaf, stem, spike, collar and root. In this paper, we report the consequence of priming in modulating *Piper nigrum* defense against *Phytophthora capsici*. Glycol Chitosan (GC) was used to infiltrate detached leaves of mature *P. nigrum* plants. It was observed that pre-treatment of GC for 24 hours resulted in significant reduction of disease symptoms in infected leaves, as evidenced by the marked decrease in the size of lesions, and also delayed the appearance of symptoms up to 72 hpi. Experiments repeated in *P. nigrum* seedlings under controlled growth conditions indicate that delayed disease symptoms of GC pre-treated leaves do not spread to healthy uninfiltrated leaves suggesting a priming-associated systemic defense response. An ROS-mediated manifestation of Hypersensitive Response (HR) induced by Chitosan was also evident in pre-treated leaves. A corresponding visual indication of increased lignification was observed, which correlated with an enhanced lignin content of GC-treated leaves. Enhanced callose deposition was also apparent in GC infiltrated leaves, establishing a stimulatory effect of GC in triggering HR through ROS production, enhanced lignification and callose deposition. Key genes of the core phenylpropanoid and isoprenoid pathways along with major defense signalling pathway genes of *P. nigrum*, including pathogenesis-related genes and hormone signalling genes showed significant transcript enrichment consequential to GC treatment. A significant quantitative enhancement in Piperine content was evident in GC-infiltrated leaves. The systemic nature of priming on disease protection was established through experiments conducted in rooted cuttings monitored for 30 days after disease infection. This is the first report that provides strong molecular evidence endorsing the twofold advantage of defense priming in *P. nigrum* by improving crop protection with a concomitant enhancement in Piperine biosynthesis.

## Introduction

Black pepper (*Piper nigrum* L.), one of the oldest known spices, is a woody perennial which originated in the humid, tropical evergreen forests of the Western Ghats of India and is now grown in more than 25 countries around the world. *P. nigrum* belongs to basal angiosperms which branched off from other flowering plants before the appearance of the eudicots, or the monocots, and are hence phylogenetically unique. There are several production constraints to obtaining a sustainable yield of commercial black pepper, of which global climate change, inadequate water availability, and biotic stresses such as epidemic diseases and pests, are important challenges to be addressed (Krishnamoorthy and Parthasarathy, 2009). *Phytophthora capsici* is the major oomycete pathogen responsible for the devastating ‘quick wilt’ or ‘foot rot’ disease affecting black pepper. The pathogen infects the leaves, stems, and roots of cuttings in the nursery and pepper plantations. Foliar treatment and soil drench method of treatments with the contact fungicide Bordeaux mixture is effective in controlling *P. capsici*, but leaching out and loss of contact fungicide consequent to heavy rainfall has led to the adoption of systemic fungicide treatment measures including the use of Metalaxyl and its combinatorial use with other contact fungicides (Anandaraj and Sarma, 1995) (Satyagopal et al., 2014). However, long-established negative implications of chemical fungicides on environmental or human health (Hausbeck and Lamour, 2004), phytotoxicity (Foster and Hausbeck, 2010), and fungicide resistance (Lamour and Hausbeck, 2000) have evoked the search for ‘cleaner’ biocontrol strategies for crop protection. Consequently, *Trichoderma* and many types of bacteria including Actinomyces spp, *Burkholderia cepacian*, and species of *Pseudomonas and Serratia* have been effectively used alone or in combination with the chemical fungicides to control *P. capsici* (Özyilmaz and Benlioglu, 2013). Such bio-stimulants can “prime” plants’ defenses by enhancing their responsiveness to biotic stressors through an initial trigger of a minor defense response that increases the plant’s ability to defend itself against future antagonists. ‘Defense priming’ increases the responsiveness of the host immune system (basal defense) in response to selected environmental signals (Mauch-Mani et al., 2017). Basal resistance by itself is too weak to protect against virulent pathogens since it constitutes only a residual level of resistance after immune suppression by the pathogen. However, priming-inducing stimuli can render basal resistance more effective, particularly when the accelerated defense response precedes immune suppression by the invading pathogen (Ahmad et al., 2010). The fitness costs of priming are lower than those of constitutively activated defense suggesting that priming functions as an ecological adaptation of the plant to respond faster to a hostile environment (van Hulten et al., 2006) (Singh, 2021).

Stimulus from pathogens, beneficial microbes, insects, chemical elicitors, and abiotic stress bring the plant into the priming phase, which is marked by the accumulation of calcium, tricarboxylic acids, reactive oxygen species, hormone conjugates, amino acids, sugars, and post-transcriptional modifications and activation of defense-related genes (Chassot et al., 2008) (Beneloujaephajri et al., 2013). Such metabolic imprints may prime the metabolic responses of plants to subsequent environmental stresses (Schwachtje et al., 2019).

Priming agents may be live organisms, chemicals, or components thereof, and can be applied to various tissues and at diverse developmental stages (Westman et al., 2019). Among many known elicitors that prime plant defense, chitin from crustacean shells and its deacetylated form chitosan, are known to prime and induce plant defense against pathogens (Lopez-Moya et al., 2017). Chitin-specific receptors on plant cell surface recognize pathogen-encoded pathogen-associated molecular patterns (PAMPs) and activate PAMP-triggered immunity (PTI) that can induce defense responses against potential fungal, bacterial, and viral pathogens (Pusztahelyi, 2018). However, because of its high crystallinity, commercially available bulk Chitin is not soluble in most common solvents. GC, a water-soluble chitosan derivative with hydrophilic ethylene glycol branches is, on the other hand, biocompatible and non-cytotoxic.

Most of the studies pertaining to molecular mechanisms of priming have been conducted on model plant pathosystems, but a deeper understanding of the discrete molecular imprints of priming in a non-model pathosystem like *P. nigrum* x *P. capsici* is crucial for the successful application of this approach in crop protection as priming mechanism is host dependent.

The first *P. nigrum* reference genome data and its assembly were reported recently (Hu et al., 2019), providing an evolutionary perspective and insight into the metabolic processes and molecular basis of species-specific Piperine biosynthesis. Very recently, a comprehensive web-genomic resource of black pepper was developed by (Negi et al., 2022), which catalogues genome-wide deep mining of SSR markers from the genome assembly of black pepper. Studies on the identification of putative biotic and abiotic stress-associated genes by transcriptomics sequencing approaches (Hao et al., 2016) (Negi et al., 2021), have produced important basic information and serve as valuable resources for future breeding programmes. There is a single report of the favorable effect of chitosan treatment in *P. nigrum* seedlings which promoted plant growth and reduced disease severity of the stunting disease caused by Cucumber mosaic virus (CMV) in Indonesia (Uge et al., 2018). However, no reports are available on studies that systematically explore the molecular mechanisms of defense priming in *P. nigrum*.

Soil treatment with lime which consists of minute fragments of the chitin-rich exoskeleton of marine organisms is frequently practiced in black pepper plantations to ameliorate quick wilt caused by *P. capsici*. Our lab has been working on *Piper* species and its interaction with *P. capsici* for the last few years (Mani et al., 2012) (Krishnan et al., 2015) (Mani and Manjula, 2020) (Geetha et al., 2021) to cite a few, and efforts were directed towards generating proteome and transcriptome data in *P. nigrum*, wherein we optimized a unique transcriptome-based proteomics strategy for deciphering the host immunity of *P. nigrum.* The proteome’s differential response led us to hypothesize that innate immunity could be a key mechanism targeted and manipulated by the hemibiotroph, *P. capsici*, facilitating its early compatible interaction with *P. nigrum* (Mahadevan et al., 2016). Our preliminary observation that pre-treatment of leaves with GC, prior to infection significantly delay disease symptoms in *P. nigrum*, urged us to further explore the potential role of GC as an elicitor of innate immunity in *P. nigrum* to defend against *P. capsici* infection. This paper provides molecular evidence based on experiments conducted on detached leaves, seedlings as well as cuttings to support this hypothesis. This is the first empirical data supporting the benefits of priming in *Piper nigrum*, for which detailed studies on stress signalling and host defense responses are limited due to the inadequacy of molecular resources and annotated functional data, despite the global importance of the crop as the ‘King of Spices’.

## Materials and methods

### Plant material and *Phytophthora capsici* culture

*Piper nigrum L.* variety- Panniyur I was maintained in the greenhouse at Rajiv Gandhi Centre for Biotechnology, Thiruvananthapuram. Second and third leaves from mature plants, growing under uniform conditions, were utilized for the leaf assay. For the establishment of *P. nigrum* seedlings, mature red berries from *P. nigrum* were collected from the greenhouse, washed and immersed in sterile distilled water for 24 hours to remove the outer covering. The seeds thus obtained were sown in autoclaved sterile soil (30% garden soil + 70% soil rite) in pots inside the growth chamber (Conviron, Germany), maintained at 70% RH and 26 ± 2°C under 12h photoperiod adjusted with white fluorescent tube light. 10-month-old seedlings were taken for further experiments. A field study was conducted in mature rooted cuttings of *P. nigrum*, sourced from the College of Agriculture, Vellayani, and maintained in the greenhouse of Rajiv Gandhi Centre for Biotechnology. The strain of *Phytophthora capsici*, virulent to *Piper nigrum L* (RGCB0451) was maintained by continuous sub-culture on potato dextrose agar (PDA) medium (HiMedia, India) at 28°C.

### Elicitor treatment

Glycol chitosan (GC) (Sigma Aldrich, USA), dissolved in sterile distilled water at a concentration of 1 mg/ml was used for the experiments. It was administered to the abaxial side of the leaf lamina of detached *P. nigrum* leaves and seedlings by infiltration using a needle-less syringe and incubated for 24 hours. A minimum of ten leaves were infiltrated each time, for all the experiments. Leaves infiltrated with distilled water were used as the mock control wherever applicable.

### Infection assay

Detached leaves from *P. nigrum* plants were thoroughly cleaned and inoculated with *P. capsici* using mycelial agar plug inoculation method (Krishnan et al., 2015). The abaxial side of the second and third leaves from a minimum of ten *P. nigrum* plants was pinpricked in at least 6-8 points and inoculated with *P. capsici* mycelial plug (7 mm in diameter) collected from the growing edge of 5-day-old cultures. The leaves were then placed in plastic square petri-plates at 27 ± 1°C, with moist cotton plugs to maintain humidity. After the incubation period, the agar plugs from the site of infection were removed. Leaf discs of 7.0 mm diameter were procured using a paper hole puncher from the inoculation site of all the leaves and immediately frozen in liquid nitrogen (Zuluaga et al., 2016) prior to total RNA and genomic DNA isolation. For the infection assay of seedlings and cuttings, the abaxial side of the leaves of *P. nigrum* was inoculated with *P. capsici* mycelial plugs as previously described. Glycol chitosan (1mg/ml) was infiltrated using a needle-less syringe, 24 hours prior to infection.

### Microscopic analysis of the progress of the infection using Trypan blue staining

Trypan blue staining was performed to visualize the pathogen and to assess the progression of *P. capsici* infection at different time points in detached leaves of *P. nigrum* (before and after GC treatments). The staining protocol described by (Chung et al., 2010) was followed, with slight modifications. Briefly, the leaf discs were immersed in acetic acid: ethanol (1:3) solution for 24 hours, followed by incubation in an acetic acid: ethanol: glycerol (1:5:1) solution for nearly 12 hours. The discs were then incubated for 6-12 hours in a staining solution of 0.01% Trypan Blue in lactophenol and washed twice with 60% glycerol. The stained samples were then placed on glass slides with 60% glycerol and examined under a Nikon Eclipse-Ni microscope with magnification 20X.

### Measurement of lesion size

*P. nigrum* detached leaves were infected with *P. capsici* 24 hours post infiltration with water (mock control) and GC along with un-infiltrated *P. nigrum* leaves (control). The diameter of the circular lesion (mm) was recorded at different time points (6, 12, 24, 48 & 72 hours) until the leaves lost their vigour (72 hours). The experiment was performed 5 times with 8-10 leaves per treatment each time. The lesion area was calculated according to the formula, area=π*r^2^/4 (mm^2^).

### Aniline Blue staining for callose deposition

Visualization of callose deposition was done using Aniline Blue staining according to the protocol described by (Jin and Mackey, 2017). Leaf discs were destained in lactophenol solution overnight, until almost transparent. The discs were then washed with 50% ethanol for 5 minutes, followed by water for 10 minutes and immersed in the staining solution (150 mM K_2_HPO_4_ + 0.01% aniline blue) for 1 hour. Images were captured using Leica SP2 Confocal Microscope with magnification 10X.

### Visualization of ROS

2′− 7′- dichlorodihydrofluorescein diacetate (H_2_DCFDA) staining was used to detect ROS production in detached *P. nigrum* leaves. The protocol followed was as described by (Juárez et al., 2015), with slight modifications. Briefly, sections of *P. nigrum* experimental leaf samples were incubated in 50mM H_2_DCFDA under dark conditions for 15 minutes. The sections were washed twice in milliQ water, mounted on glass slides and covered with a cover slip. The samples were then examined using Leica SP2 Confocal Microscope under 20X magnification.

### Weisner staining for lignin

The degree of lignification during plant-pathogen interaction as well as after glycol chitosan treatment was observed using Wiesner staining (Phloroglucinol-HCl) (Pradhan Mitra and Loqué, 2014). Briefly, 3% phloroglucinol was prepared by dissolving 0.3g phloroglucinol in 10ml absolute ethanol. Weisner stain was prepared by mixing two volumes of 3% Phloroglucinol with one volume of Hydrochloric acid. The leaf sections were immersed in the stain for 5 minutes at room temperature. The sections were immediately transferred to a glass slide, covered with a cover slip, and observed under bright field lighting in a Nikon Eclipse-Ni microscope under 4X magnification.

### Quantification of lignin

Acetyl bromide assay was carried out for the quantification of lignin in control, *P. capsici* infected, water infiltrated (mock control) and GC infiltrated detached *P. nigrum* leaves. Protein-free cell wall preparation, digestion & solubilization was performed as per the protocol by (Moreira-Vilar et al., 2014). The lignin concentration in all the samples was calculated according to (Fukushima and Kerley, 2011).

### Selection of contigs for the present study

The gene candidates (**Supplementary Table 1**) were selected from a transcriptome library of *P. nigrum* that we had generated earlier from control (untreated leaf of *P. nigrum*) and GC-treated (1mg/mL for 24 hours) detached leaves of *P. nigrum* (Accession: SRX1715099). The raw sequence data are deposited in the NCBI Sequence Read Archive (SRA) under the BioProject accession number PRJNA318916.

### Mapping of assembled transcripts to reference genome

The final assembled transcripts in fasta format (Accession: SRX1715099) and the *P. nigrum* reference genome (PRJNA529758) (Hu et al., 2019), served as the framework for the mapping and alignment of transcripts. The transcripts were mapped to the reference genome using the Magic-blast method, which functions well in the mapping of long reads and the identification of introns despite being single pass and aligning RNA to the genome without knowledge of a transcriptome annotation (Boratyn et al., 2019). In order to provide a comprehensive understanding of the visualization of the transcript data across the entire *P. nigrum* genome, a Circos plot was generated using Circos package (Krzywinski et al., 2009), version 0.69-9.

### Genomic DNA extraction and quantification of absolute biomass of *Phytophthora capsici*

Total genomic DNA was extracted from the pooled sets of control leaves, leaves infected with *P. capsici* and leaves pre-treated with GC followed by pathogen infection using NucleoSpin^®^ Plant II Genomic DNA Extraction kit (Macherey-Nagel). The DNA-based Real-Time PCR (qPCR) technique (Qi and Yang, 2002) was used to determine the relative growth and absolute biomass of *P. capsici* before and after glycol chitosan and mock control treatments. *P. capsici-*specific 28S rRNA sequence was used to design the primer using the Primer3Plus online tool (https://primer3plus.com/cgi-bin/dev/primer3plus.cgi). The primer was checked for primer dimer and multiple product formation by analyzing the melt curve. qPCR analysis was carried out using SYBR Premix Ex Taq II (Takara) on QuantStudio 5 Real-Time PCR system (Applied Biosystems). The PCR volumes were set to 20 μl and contained 10 μl SYBR Green PCR Premix (Takara), 2 μl DNA template, 0.4 μl ROX Reference Dye (50X), 10 μM each of the corresponding forward and reverse primers and 6 μl sterile nuclease-free water. The PCR conditions were 50°C for 2 min, 95°C for 10 min, and 40 cycles of 15 s at 95°C and 1 min at 60°C. All reactions were performed in triplicates, with three biological replications. The comparative C_T_ method (2^−ΔΔCT^ method (Schmittgen and Livak, 2008) was utilized for the quantitative gene expression studies. *Piper nigrum* 18S rRNA was used for the normalization of expression data. The specific primers used for the study are given in **Supplementary table 1**.

### RNA extraction and cDNA synthesis

Total RNA from each treatment and control leaf discs was isolated separately using Nucleospin^®^ RNA plant (Macherey-Nagel) kit. This was followed by the first-strand cDNA synthesis using 1 µg of total RNA from each sample using PrimeScript™ RT Reagent Kit (Perfect Real Time) following manufacturer’s instructions.

### qRT-PCR analysis of the candidate genes

Primer sequences for qRT-PCR analysis of the selected genes were designed using the Primer3Plus online tool (https://primer3plus.com/cgi-bin/dev/primer3plus.cgi). The primer sequences for all the genes are given in **Supplementary Table 1**. For expression analysis of the genes, qRT-PCR analysis was carried out as described in the previous section. All reactions were performed in triplicates and repeated thrice (biological replicates). The comparative C_T_ method [2^−ΔΔCT^ method (Schmittgen and Livak, 2008),] was utilized for the quantitative gene expression studies. For normalization of the expression data, *P. nigrum* 18S rRNA was used.

### Gene ontology analysis

Genes which showed significant upregulation in response to GC treatment from the qRT-PCR expression studies were selected for Gene Ontology analysis using PANTHER (Protein Analysis Through Evolutionary Relationships) database version 16 (Mi et al., 2021). The input gene list was classified against *Arabidopsis thaliana* gene database followed by a statistical over-representation test. Biological processes showing fold enrichment were selected after false discovery rate (FDR) correction.

### HPLC analysis of *Piper nigrum* leaves

*P. nigrum* experimental leaf samples were air-dried and powdered. 10 ml of 100% HPLC-grade methanol was added to 1g powder of each experimental sample, sonicated for 2 minutes and centrifuged for 2 minutes at 11,000 rpm. The supernatant thus obtained was used for further analysis. Rapid Analytical RP-HPLC analysis was performed on a Shimadzu CLASS-VP V6.14 SP2 system using a C18 column, maintained at room temperature, for the determination of Piperine in the experimental samples following the previously reported method (Upadhyay et al., 2013). Acetonitrile: Water: Acetic acid (60:39.5:0.5) was used as the mobile phase, with a flow rate of 0.5 ml/min, passing through a 500 μl loop. The mobile phase was filtered using a 0.45 μm Millipore filter, followed by sonication for 30 minutes, before use. Piperine was identified at 343 nm (12.9 minutes retention time) in the detector attached to the HPLC system based on co-chromatography with standard Piperine (Sigma-Aldrich, USA). Quantification of Piperine in the experimental samples was done by comparison with the standard curve plotted using different concentrations (20, 40, 60, 80 & 100 ppm) of standard Piperine in methanol, each taken in triplicates.

### Targeted metabolomics

Approximately 750 mg of each experimental sample were extracted using 1.5ml 90% methanol. Samples were sonicated for 2 minutes and centrifuged for 5 minutes at 4000rpm. Supernatants were transferred to fresh tubes and dried under vacuum. Dried samples were reconstituted in 50μl of 100% methanol from which 10μl was taken for each injection. Piperine and major classes of secondary metabolites-the flavonoids and coumarin were targeted to quantify their accumulation in response to *P. capsici* infection and glycol chitosan treatment. 1mg of each standard was weighed and dissolved in 1 ml of 100% methanol to get a final concentration of 1 mg/ml. 16 standard pools were prepared in 90% methanol and serially diluted in 100% methanol to get an 8-point calibration curve. 10μl of each sample was injected for the analysis. LC separation was performed on a Dionex Ultimate 3000 UHPLC system with the following parameters: Column - Phenomenex, Jupiter, 5µ, C18, 300A, 150mm x 4.6mm 5-micron, Mobile Phase A - 10mM Ammonium Acetate in Water (0.1% FA), Mobile Phase B - Acetonitrile (0.1% FA), Flow Rate - 0.4ml/min, Column Oven - 40°C, Auto-sampler Temp. - 10°C, Injection Volume - 10µl, Run Time - 28mins, Gradient - 0-2mins:0.5%B, 2-3mins:0.5-10%B, 3-13mins:10-25%B, 13-22mins:25-100%B, 22-23.1mins:100-0.5%B, 23.1-28mins:0.5%B. MS analysis was performed in ThermoFisher Q Exactive with a (+ve) Spray Voltage of 4000V and (-ve) Spray Voltage of 2500V, Vaporizer temp - 280°C, Sheath gas flow rate - 30Arb, Aux gas flow rate - 10Arb, Injector settings - 0-5mins: waste, 5-26.5mins: load, 26.5-28mins: waste.

### Field study

The basal leaf of *P. nigrum* plants were infiltrated with GC, followed by inoculation with *P. capsici* after 15 & 30 days of GC application in both infiltrated and uninfiltrated leaves of each plant. 100 plants were studied under each treatment and the plants were observed for infection progression up to 4 weeks. The disease incidence with and without GC treatment was calculated according to the formula:


Disease Incidence (DI) =No:of infected plantsTotal no:of plants×100

### Statistical analysis

The mean, standard deviation, and standard error of the mean (SEM) were calculated for all the experiments, and data are presented as mean ± SEM. All statistical analysis of data was carried out using the software GraphPad Prism 9. One-way ANOVA (p ≤ 0.05) was used for comparison among treatments in all the experiments except in expression analysis of hypersensitive response genes where a two-way ANOVA (p ≤ 0.05) was used. Tukey’s *Post hoc* multiple comparison test (p = 0.05) was used to determine the significant difference among groups.

## Results

### *Phytophthora capsici* produces early symptoms of infection and colonizes *Piper nigrum* leaves within 24hpi

**Figure 1:**
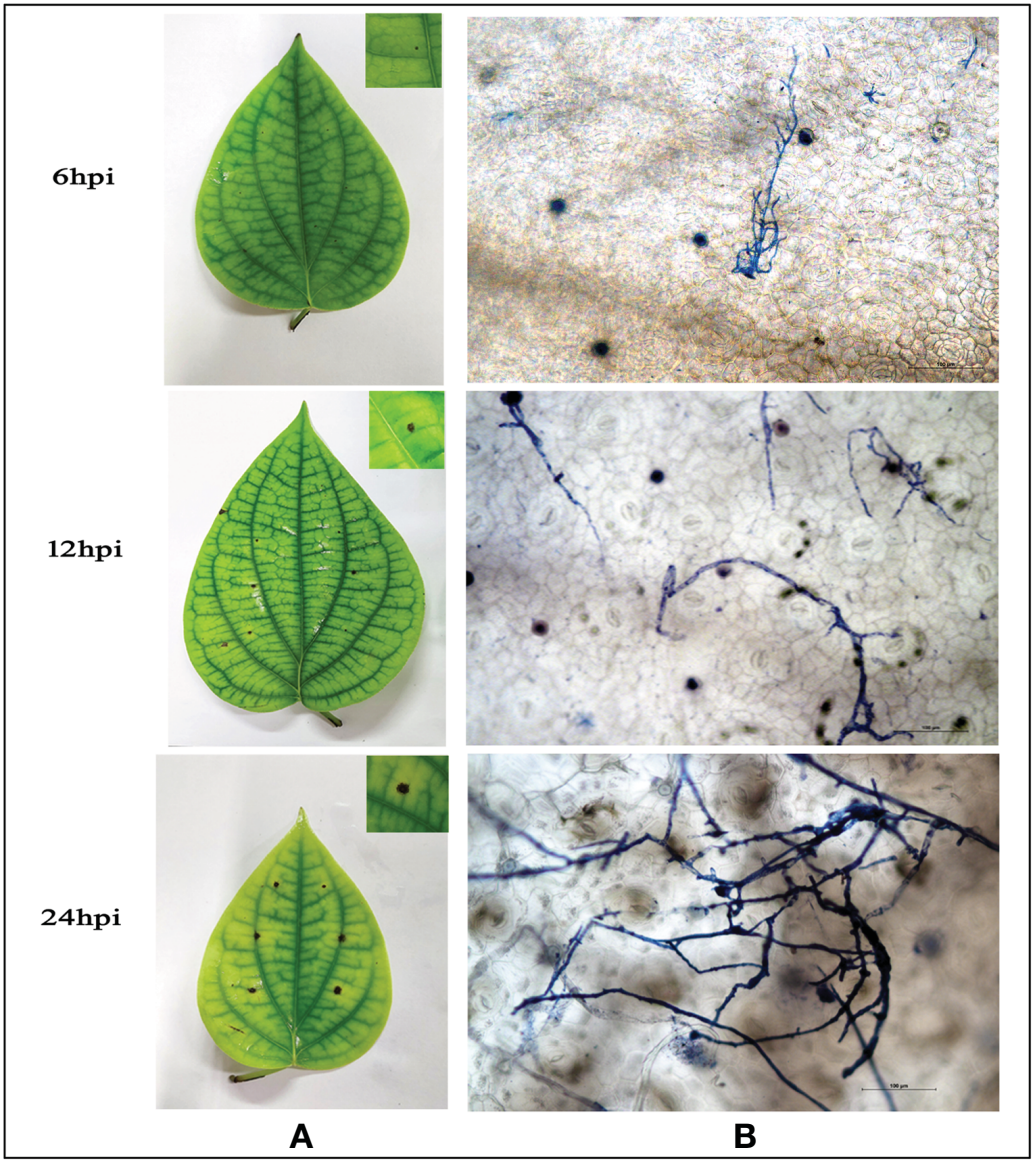
**(A)** Macroscopic observation of *P. capsici* infection progression in detached *P. nigrum* leaves at 6,12, and 24 hpi **(B)** Microscopic observation of *P. capsici* infection progression in detached *P. nigrum* leaves at 6, 12, and 24 hpi using trypan blue staining, visualized under 20X magnification, Scale bar: 100μm.

A virulent strain of *P. capsici* (RGCB0451) shows hyphal growth initiation at 6hpi on detached leaves of *P. nigrum* and profusely proliferates to colonize the leaf surface producing progressing necrotic lesions within 24hpi (**Figure 1A**). Microscopic observations showed that the infection starts as early as 6 hpi, where the pathogen is seen entering the leaf tissue mostly through stomatal openings. The infection progresses with time and from 24hpi, it could be observed that there is extensive colonization of *P. capsici* in the leaf tissue along with necrosis (**Figure 1B**). Due to the manifestation of symptoms denoting complete host susceptibility within 24hpi, observations of results of all further experiments were consistently recorded at 24hpi.

### Foliar pre-treatment with glycol chitosan reduces the growth of *Phytophthora capsici* in *Piper nigrum* leaves

**Figure 2:**
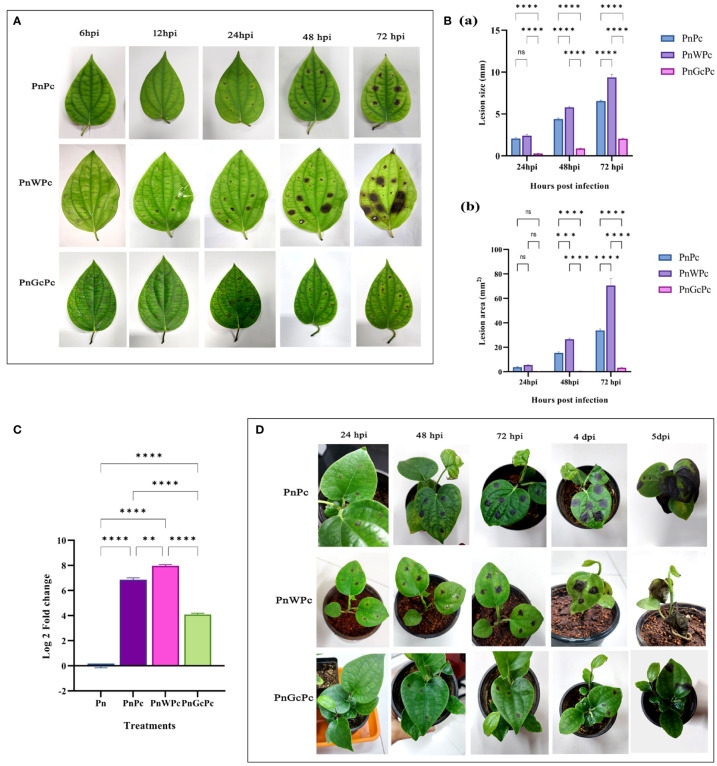
*P. capsici* infection progression in *P. nigrum* after specific treatments **(A)** *P. nigrum* detached leaves (PnPc: *P. nigrum* infected with *P. capsici*, PnWPc: *P. nigrum* infected with *P. capsici* 24h post infiltration with water & PnGcPc: *P. nigrum* infected with *P. capsici* 24h post infiltration with GC) at 6, 12, 24, 48 & 72 hpi **(B)** Comparison of *P. capsici* infection lesion size (a) Lesion diameter (mm) (b) Lesion area (mm^2^), among treatments at 24, 48 & 72 hpi **(C)** Quantification of the relative growth & absolute biomass of *P. capsici* in detached *P. nigrum* leaves using qRT-PCR with *P. capsici* specific 28S rRNA primers. (Pn: *P. nigrum* control, PnPc: *P. nigrum* infected with *P. capsici*, 24 hpi, PnWPc: *P. nigrum* infected with *P. capsici* 24h post infiltration with water, 24 hpi, PnGcPc: *P. nigrum* infected with *P. capsici* 24h post infiltration with GC, 24 hpi), **(D)** *P. capsici* infection progression in control (Pn), water infiltrated (PnW), and GC infiltrated *P. nigrum* (PnGc) seedlings at 24hpi, 48hpi, 72hpi, 4dpi & 5dpi. Comparative gene expression was calculated according to the 2^−ΔΔCT^ method (Schmittgen and Livak, 2008). One-way ANOVA was performed to compare among treatments. Values are means of triplicates. Data are expressed as Mean ± SEM. p ≤ 0.01, 0.001, 0.0001 are represented by **, ***, **** respectively and p ≥ 0.05 is represented by ns (not significant).

Treatment of detached leaves of *P. nigrum* with GC (1mg/mL), 24 hours prior to *P. capsici* mycelial plug infection resulted in significant reduction in disease symptoms, as evidenced by a marked decrease in the size of lesions (**Figures 2A, B**) and delayed the appearance of necrotic symptoms up to 72 hpi (**Figure 2A**, right extreme panel). This observation is further supported by the corresponding reduction of pathogen DNA at all stages of infection in GC pre-treated leaves, in comparison to infected control (untreated) leaves and mock(water) infiltrated infected leaves (**Figure 2C**). After 72h the detached leaves lost their robustness and hence protective role of GC was consistently tested in detached leaves only up to 72hpi. In seedling leaves, infiltration of GC delayed the appearance of disease symptoms and slowed down infection progress (**Figure 2D**). The infected leaf in GC-treated seedlings survived up to 10 days, whereas in the control and mock control seedlings, the leaves withered off and wilted right after the 5^th^ day. A noteworthy observation was that, there was no sign of infection spreading to fresh un-infiltrated leaves in GC-treated seedlings even after 4 weeks, in contrast to the control and mock control plants which succumbed to the infection completely within 10 days,

### *Piper nigrum* transcriptome maps to published *Piper nigrum* whole genome (PRJNA529758) with high significance

**Figure 3:**
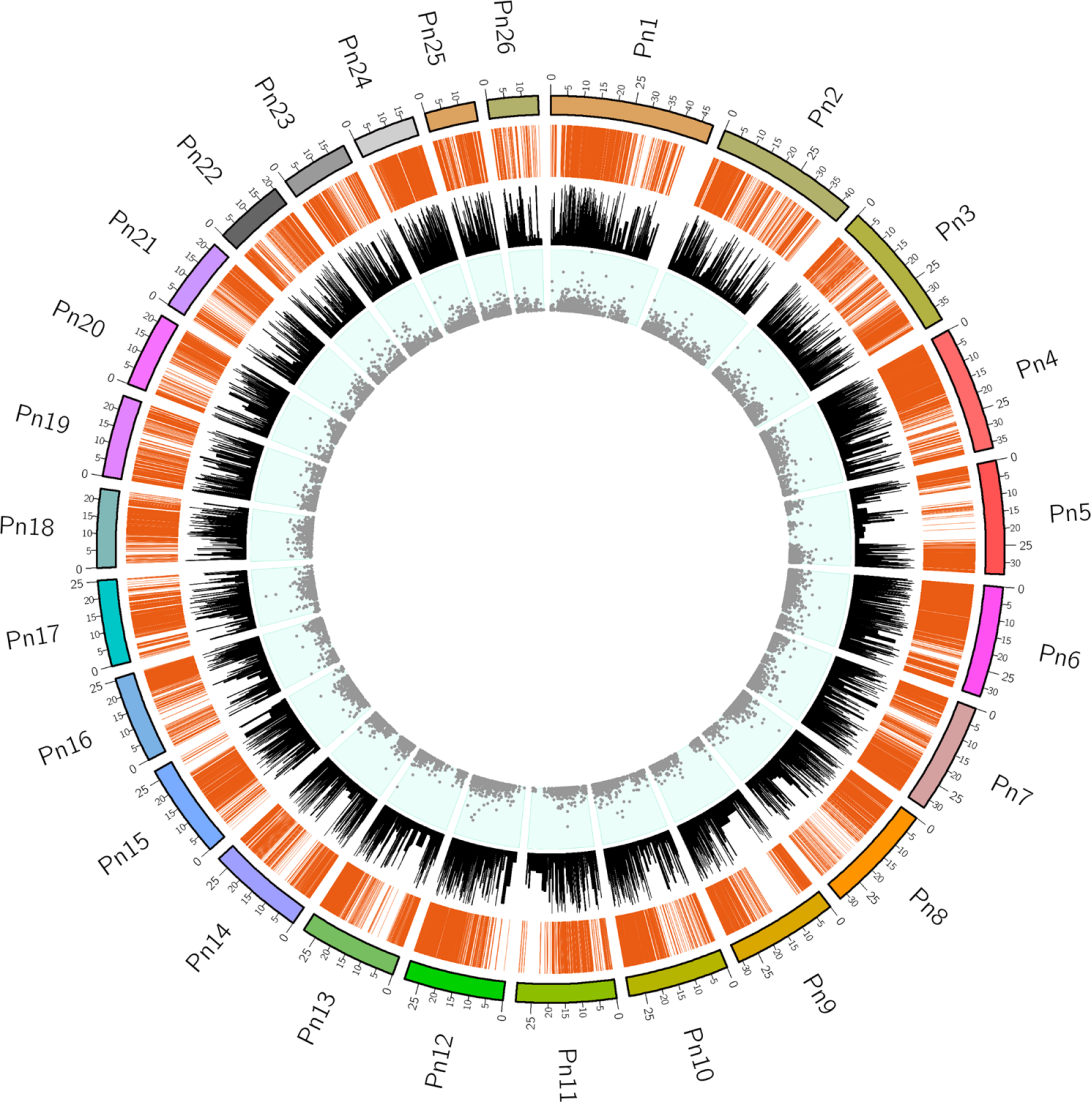
The Circos plot for the transcriptome dataset mapped to the *Piper nigrum* genome. Pn1-Pn26 indicate the chromosomes in *P.nigrum.* Connectors indicate transcription coverage of the actively transcribed genes mapped in the diagonal line region of the plot. An increased frequency of reads aligned denotes greater transcription activity when compared to other regions (outermost track). The sequence identity score with the *Piper nigrum* genome for the elevated expression transcripts aligned to a locus in comparison to the rest of the genome is shown in bar charts (middle track). The data points correspond to the composite scores of the transcripts aligned to the chromosome locus shown as a scatter plot (innermost track).

To assess the extent of transcript coverage, we chose the whole genome of *Piper nigrum* (PRJNA529758) which was matched to the transcripts generated using the blast read aligner Magic-blast. The dataset provided consists of genome and RNA-seq, in which the reads were aligned using a version of magic-blast. By screening assembled transcripts, it was possible to determine the level of complete transcript coverage and this was found to be 36269 mRNAs produced by coding genes, which included the fully covered (represented by 11870 mRNAs), and > 90% coverage (represented by nearly 31936 transcript genes) on the *Piper nigrum* genome. The Circos plot in **Figure 3** provides a genome-wide view of the transcript coverage of *P. nigrum*. The most striking feature of the Circos plot is the strong connectors indicating transcription coverage of the active genes mapped in a diagonal line of the plot and are characterized by long transcribed sequences, the increased frequency of reads aligned denotes a greater transcription activity when compared to other regions in this plot. The bar charts represent the identity score for the transcripts aligned to a locus compared to the rest of the genome. The scatter plot in the Circos plot represents the composite score of the transcript alignment, with data points proportionate to the composite score (**Figure 3**). The aligned transcriptome (Accession: SRX1715099) was the source of all the genes selected for the present study. Key genes of the defense pathways representing those regulating ROS generation, hypersensitive response, lignification, callose deposition, phenylpropanoid, pathogenesis-related and phytohormone signalling were selected for further expression analysis (**Supplementary table 1**).

### GC induces ROS generation and hypersensitive response

**Figure 4:**
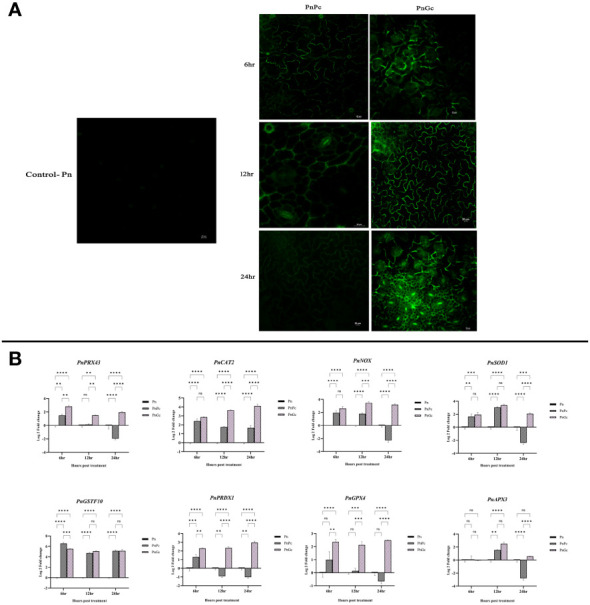
Visualization of ROS production using DCFDA staining in detached leaves of *P. nigrum* under 20X magnification. The green fluorescence in the figure is indicative of the ROS production after different treatments. Scale bar: 20μm **(A)** Pn: Control *P. nigrum*, PnPc: *P. nigrum* infected with *P. capsici* at 6, 12 & 24hpi, PnGc: *P. nigrum* infiltrated with GC at 6, 12 & 24h **(B)** Expression analysis of hypersensitive response genes (*PnPRX43:* Peroxidase*, PnCAT2*: Catalase, *PnNOX:* NADPH Oxidase, *PnSOD1:* Superoxide dismutase, *PnGSTF10*: Glutathione S-Transferase, *PnPRDX1:* Peroxiredoxin, *PnGPX4*: Glutathione peroxidase, *PnAPX3:* Ascorbate peroxidase) under different treatments (*P. nigrum* control (Pn), *P. nigrum* infected with *P. capsici* (PnPc*)*, *P. nigrum* infiltrated with GC (PnGc) at 6,12 & 24h post treatments). Comparative gene expression was calculated according to the 2^−ΔΔCT^ method (Schmittgen and Livak, 2008). Two-way ANOVA was performed to compare among treatments. Values are means of triplicates. Data are expressed as Mean ± SEM. p ≤ 0.01, 0.001, 0.0001 are represented by **, ***, **** respectively and p ≥ 0.05 is represented by ns (not significant)..

The burst of ROS as a result of *P. capsici* infection (6, 12 & 24 hpi) and glycol chitosan infiltration (6, 12 & 24 hours) was observed using DCFDA fluorescence staining. At 6 & 12 h, glycol chitosan infiltrated *P. nigrum* leaves showed a relatively higher amount of fluorescence than *P. capsici-*infected leaves (**Figure 4A**). At 24 hours, fluorescence persisted in glycol chitosan-infiltrated leaves in contrast to *P. capsici-*infected leaves, which showed no fluorescence (**Figure 4A**). Comparative expression levels of the selected genes provide strong evidence of the ROS generation consequential to GC treatment (**Figure 4B**). Most of the genes including the ROS-producing (NADPHO and Peroxidase) and antioxidant genes (SOD) showed significant overexpression compared to control and pathogen-infected leaves. This localized oxidative burst implicates the possibility of an ROS-mediated manifestation of Hypersensitive Response (HR) induced by GC.

### GC pre-treated leaves show increased lignification and callose deposition

**Figure 5:**
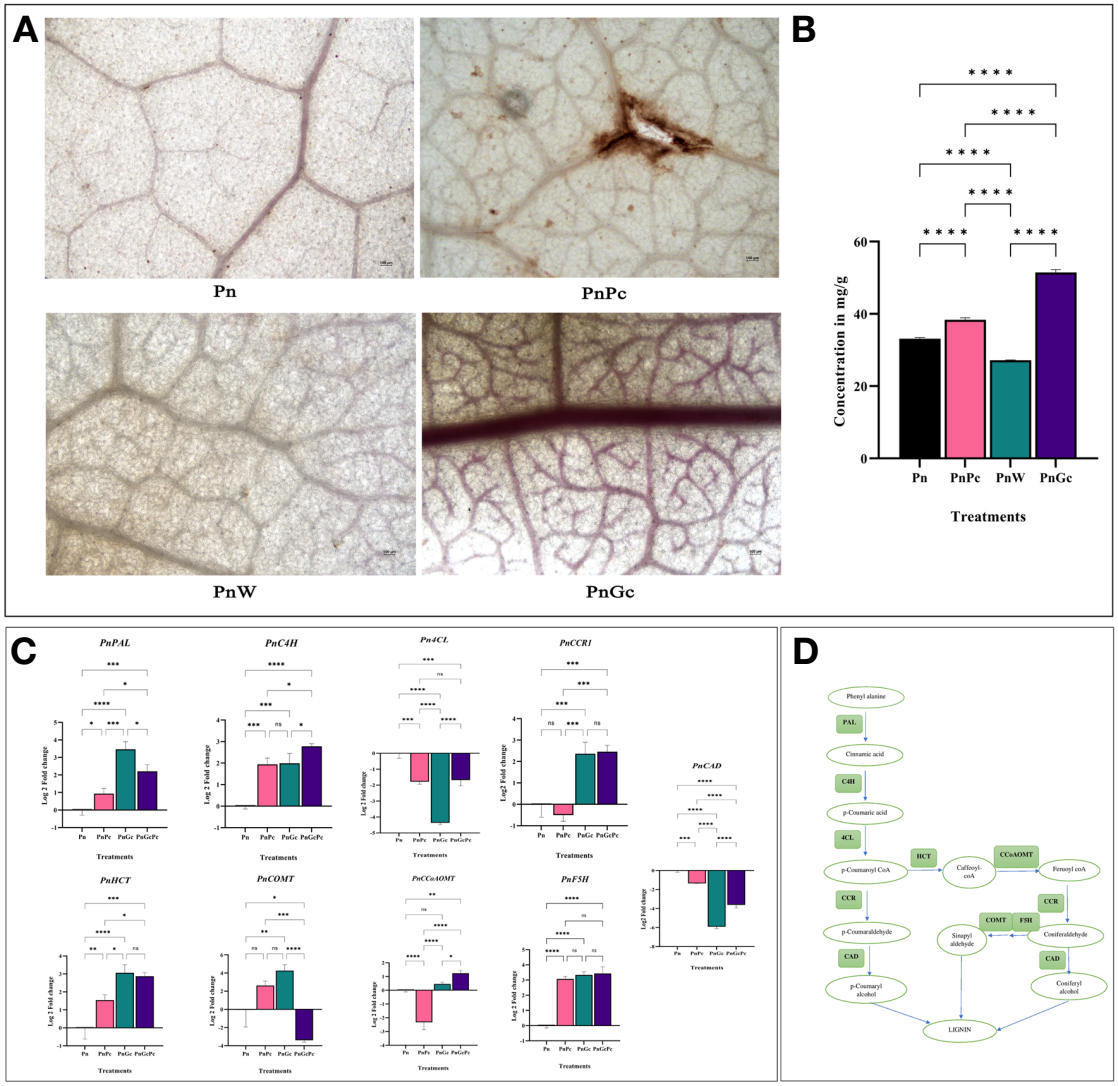
**(A)** Weisner staining to visualize lignin deposition after different treatments in detached *P. nigrum* leaves. (Pn: *P. nigrum* control, PnPc: *P. nigrum* infected with *P. capsici* 24hpi, PnW: *P. nigrum* infiltrated with water, 24h (mock control), PnGc: glycol chitosan infiltrated *P*. *nigrum* 24h), Magnification: 4X, Scale bar: 100μm; **(B)** Quantification of Lignin across different treatments by acetyl bromide method **(C)** Expression analysis of major lignin biosynthesis genes (*PnPAL:* Phenylalanine ammonia lyase*, PnC4H:* Cinnamic Acid 4- hydroxylase*, Pn4CL:*4-Coumaryl CoA Ligase*, PnCCR1:* Cinnamyl CoA Reductase*, PnHCT:* Shikimate Hydroxy Cinnamoyl Transferase*, PnCOMT:* Caffeic acid 3-O-methyltransferase*, PnCCoAOMT:* Caffeoyl CoA Methyltransferase*, PnF5H:* Ferulate 5 Hydroxylase, *PnCAD:* Cinnamyl alcohol dehydrogenase), **(D)** Simplified Lignin Biosynthesis pathway, boxes mark the genes for which expression analysis was done. Comparative gene expression was calculated according to the 2^−ΔΔCT^ method (Schmittgen and Livak, 2008). One-way ANOVA was performed to compare among treatments. Values are means of triplicates. Data are expressed as Mean ± SEM. p ≤ 0.05, 0.01, 0.001, 0.0001 are represented by *, **, ***, **** respectively and p ≥ 0.05 is represented by ns (not significant).

Lignin deposition was observed using Weisner staining (**Figure 5A**) and quantified by acetyl bromide assay (**Figure 5B**). In all samples, the brownish-pink coloration of lignified areas could be observed mostly in veins whereas, in infected and GC-treated leaves, cells from the lamina reacted with the Wiesner reagent, indicating lignin in the mesophyll. However, GC induced uniform staining and increased lignin content compared to pathogen-infected leaves. The visual manifestation of increased staining correlated well with the lignin content of leaves, which showed more than 60% increase of lignin in GC-infiltrated leaves, compared to other treatments (**Figure 5B**). These results suggest an increase in defense response modulated by enhanced lignification of affected cells.

Expression levels of key genes involved in the shikimate pathway leading to lignin biosynthesis (**Figures 5C, D**) were analyzed. The RNA levels of the genes responsible for catalyzing the final step in Monolignol biosynthesis (Cinnamyl CoA Reductase and Cinnamic Acid 4- hydroxylase) as well as two other genes involved in earlier steps of phenolic metabolism-Shikimate Hydroxy Cinnamoyl Transferase, a key metabolic entry point for the synthesis of important lignin monomers coniferyl and sinapyl alcohols, caffeoyl CoA, along with Phenylalanine ammonia lyase, the first enzyme of the phenylpropanoid pathway were monitored and compared with that of control leaves. The RNA levels of most of the genes except 4CL, and CAD showed more than 2-fold expression in GC pre-treated and GC pre-treated infected leaves compared to untreated control leaves (**Figure 5C**), while F5H was found to be upregulated across all the treatments compared to the control untreated leaves. A noteworthy observation was made concerning the regulation of CAD7, a negative regulator of plant immunity. The CAD7 homologue from the *P. nigrum* transcriptome database (Accession: SRX1715099) was selected to confirm the relevance and specificity of GC-mediated regulation of immunity. CAD7 transcripts showed more significant down-regulation in response to GC pre-treatment compared to pathogen-infected and control leaves (**Figure 5C**), which strongly indicates the positive role of GC in stimulating plant immune response.

**Figure 6:**
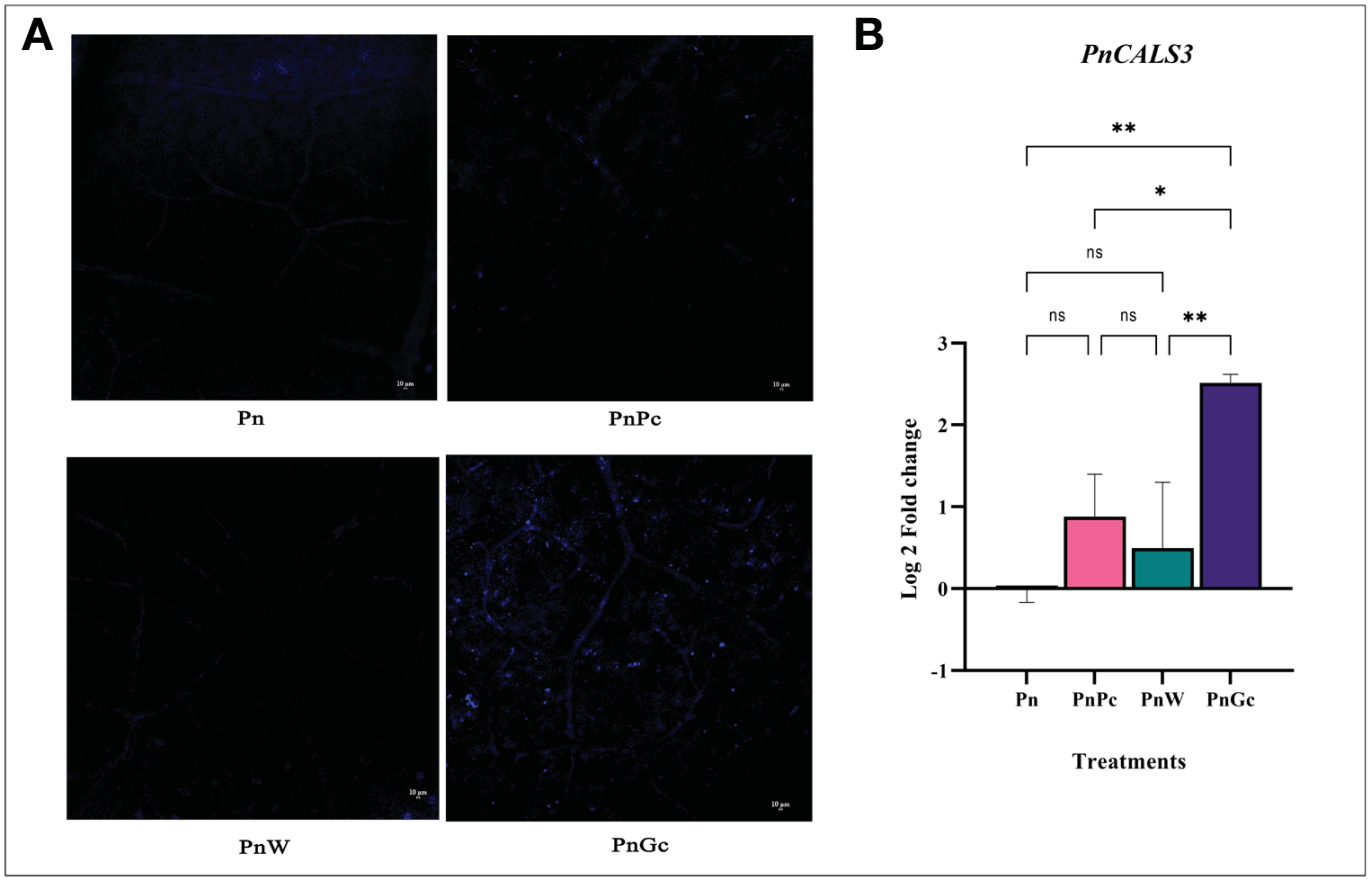
**(A)** Aniline Blue staining to visualize callose deposition after different treatments in detached *P. nigrum* leaves. (Pn: *P. nigrum* control, PnPc: *P. nigrum* infected with *P. capsici* 24hpi, PnW: *P. nigrum* infiltrated with water, 24h (mock control), PnGc: glycol chitosan infiltrated *P*. *nigrum* 24h), Magnification:10X, Scale bar: 10μm, **(B)** Expression analysis of callose synthase gene (*PnCALS3*) in detached *P. nigrum* leaves across treatments (Pn: *P. nigrum* control, PnPc: *P. nigrum* infected with *P. capsici* 24hpi, PnW: Water infiltrated *P. nigrum*, 24h, PnGc: GC infiltrated *P. nigrum*, 24h). Comparative gene expression was calculated according to the 2^−ΔΔCT^ method (Schmittgen and Livak, 2008). One-way ANOVA was performed to compare among treatments. Values are means of triplicates. Data are expressed as Mean ± SEM. p ≤ 0.05, 0.01 are represented by *, ** respectively and p ≥ 0.05 is represented by ns (not significant).

Callose deposition was visualized using aniline blue staining (**Figure 6A**) and it was observed that the GC-infiltrated leaves showed a relatively higher amount of fluorescence indicating sites of callose deposition compared to the control, water-infiltrated and *P. capsici* infected *P. nigrum* leaves. The expression analysis of callose synthase gene (**Figure 6B**) also revealed higher expression in GC-infiltrated *P. nigrum* leaves compared to control leaves, which was consistent with the staining results.

### Analysis of major phenylpropanoid and isoprenoid pathway genes

**Figure 7:**
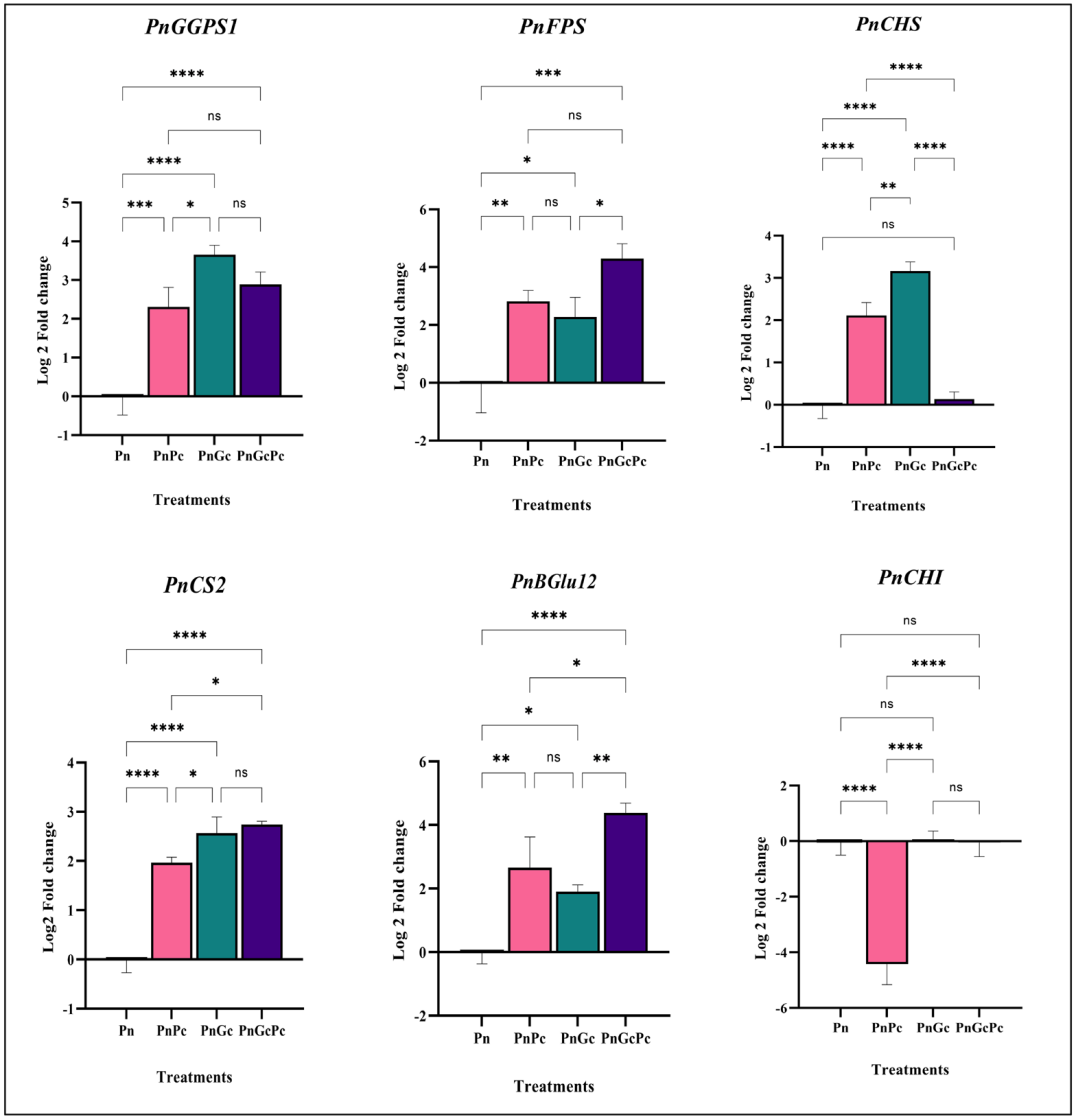
Expression analysis of major phenylpropanoid pathway and isoprenoid pathway genes *(GGPS:* Geranyl geranyl diphosphate synthase&, *FPS2:* Farnesyl diphosphate synthase, *PnCHS:* Chalcone synthase, *PnCS2*: Chorismate synthase, and *PnBGlu12:* Beta glucosidase, *PnCHI:* Chalcone isomerase) in *P. nigrum* (Pn: *P. nigrum* control, PnPc: *P. nigrum* infected with *P. capsici* 24hpi, PnGC: *P. nigrum* infiltrated with GC 24h, PnGcPc: *P. nigrum* infected with *P. capsici* 24h post infiltration with Glycol chitosan, 24hpi. Comparative gene expression was calculated according to the 2^−ΔΔCT^ method (Schmittgen and Livak, 2008). One-way ANOVA was performed to compare among treatments. Values are means of triplicates. Data are expressed as Mean ± SEM. p ≤ 0.05, 0.01, 0.001, 0.0001 are represented by *, **, ***, **** respectively and p ≥ 0.05 is represented by ns (not significant).

Data from the transcriptome (Accession: SRX1715099) we generated earlier after GC treatment of *P. nigrum* leaves, showed a differential overexpression of transcripts of phenylpropanoid genes (unpublished data), which prompted us to further establish the relevance of the in-silico differential gene expression data by *in vivo* validation approaches. The genes selected and their transcript IDs are provided in **Supplementary Table 1**. qRT-PCR analysis was performed to analyze the effects of *P. capsici* infection and GC pre-treatment (independently and in combination) on the regulation of selected genes in the core phenylpropanoid pathways which include Chalcone synthase, Chorismate synthase, Chalcone isomerase, Beta glucosidase, as well as Geranyl-Geranyl Diphosphate Synthase and Farnesyl Diphosphate Synthase, which are the 2 out of 3 important isoprenoid enzymes in plants. The transcripts of *GGPS1, CHS, CS2* & *CHI* in GC pre-treated *P. nigrum* leaves were found to be significantly upregulated when compared to control and infected leaves in contrast to *FPS* and *BGlu12* which were significantly upregulated only with respect to the control uninfiltrated healthy leaves (**Figure 7**).

### Expression analysis of key genes of plant defense signaling network

**Figure 8:**
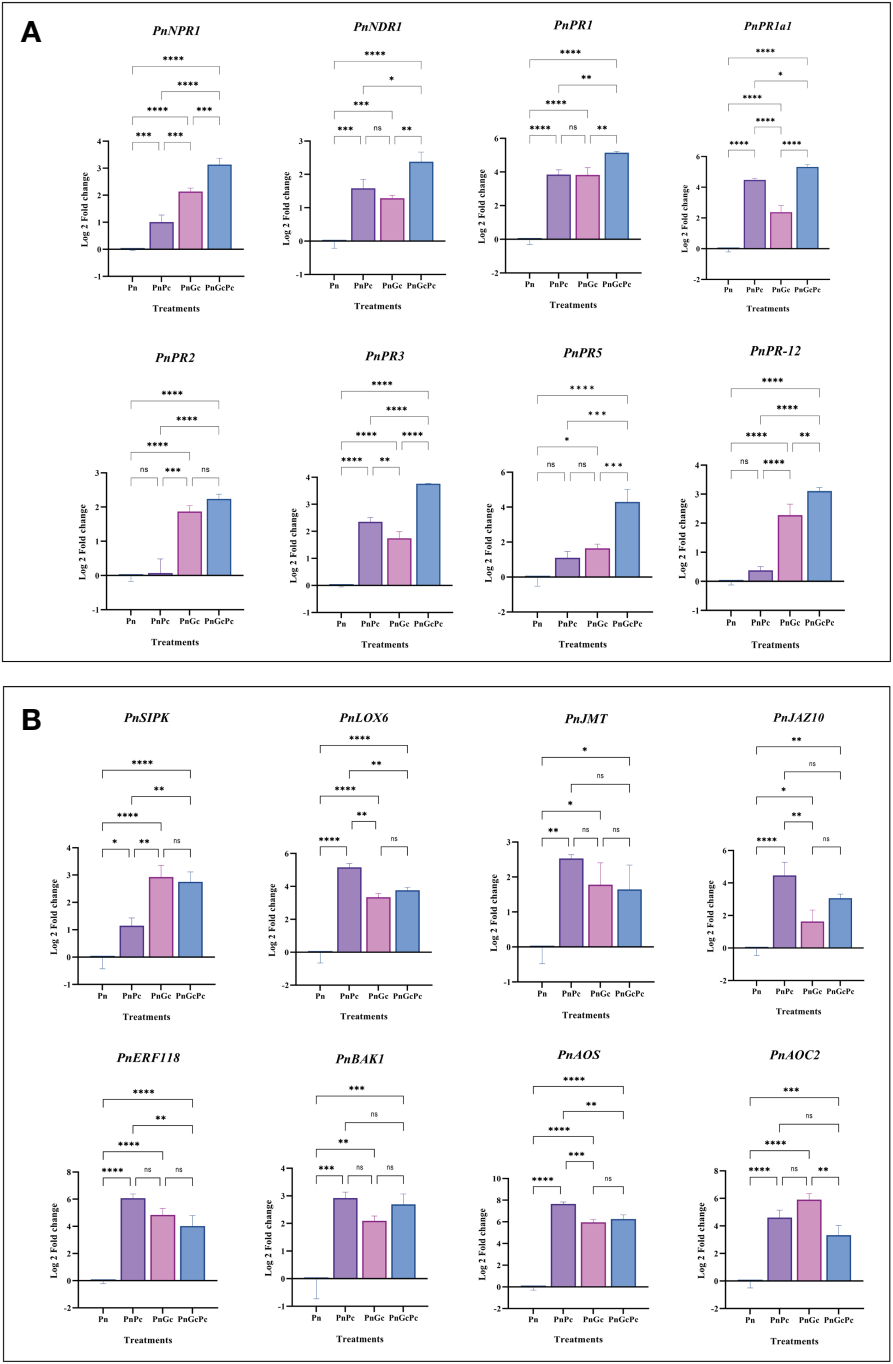
Expression analysis of plant defense signalling network genes. **(A)** Major Pathogenesis-related (PR) genes (*PnNPR1*: Non expressor of pathogenesis-related genes 1, *PnNDR1*: Non-Race Specific Disease Resistance-1, *PnPR1*: Pathogenesis-related 1, *PnPR1a1*: Pathogenesis related-1 Isoform A1, *PnPR2*: β-1,3-Glucanase, *PnPR3*: Chitinase, *PR5*: Thaumatin like*, PnPR12*: Defensin, and **(B)** Hormone signalling genes: *PnSIPK:* Putative Salicylic-induced MAPK, *PnLOX6-*Lipoxygenase 6*, PnJMT*-Jasmonate-O-Methyltransferase*, PnJAZ10*-Jasmonate zim domain protein 10*, PnAOC2*-Allene oxide cyclase 2*, PnAOS-*Cytochrome P450 allene oxide synthase*, PnERF118*-Ethylene-responsive transcription factor ERF118*, PnBAK1*-Brassinosteroid insensitive 1-associated receptor kinase1-like) in *P. nigrum* detached leaves after different treatments (Pn: *P. nigrum* control, PnPc: *P. nigrum* infected with *P. capsici* (24hpi), PnGc: Glycol chitosan infiltrated *P. nigrum* (24h), PnGcPc: *P. nigrum* infected with *P. capsici* 24h post infiltration with Glycol chitosan, 24hpi). Comparative gene expression was calculated according to the 2^−ΔΔCT^ method (Schmittgen and Livak, 2008). One-way ANOVA was performed to compare among treatments. Values are means of triplicates. Data are expressed as Mean ± SEM. p ≤ 0.05, 0.01, 0.001, 0.0001 are represented by *, **, ***, **** respectively and p ≥ 0.05 is represented by ns (not significant).

qRT-PCR analysis was performed to analyze the effects of *P. capsici* infection and GC pre-treatment (independently and in combination) on the roles of major pathogenesis-related genes (*PnNPR1, PnNDR1, PnPR1, PnPR1a1, PnPR2, PnPR3, PnPR5 and PnPR12)* and hormone signalling genes (*PnSIPK-*Putative Salicylic-induced MAPK, *PnLOX6-*Lipoxygenase 6*, PnJMT*-Jasmonate-O-Methyltransferase*, PnJAZ10*-Jasmonate zim domain protein 10*, PnAOC2*-Allene oxide cyclase 2*, PnAOS-*Cytochrome P450 allene oxide synthase*, PnERF118*-Ethylene-responsive transcription factor ERF118*, PnBAK1*-Brassinosteroid insensitive 1-associated receptor kinase1-like) in detached *P. nigrum* leaves (**Figures 8A, B**). The genes selected and their transcript IDs are provided in **Supplementary Table 1**. The expression analysis revealed that most of the genes showed considerable (more than 2-fold) upregulation in GC-treated leaves, compared to the control (**Figure 8**).

### Gene ontology analysis of significantly up-regulated genes

**Figure 9:**
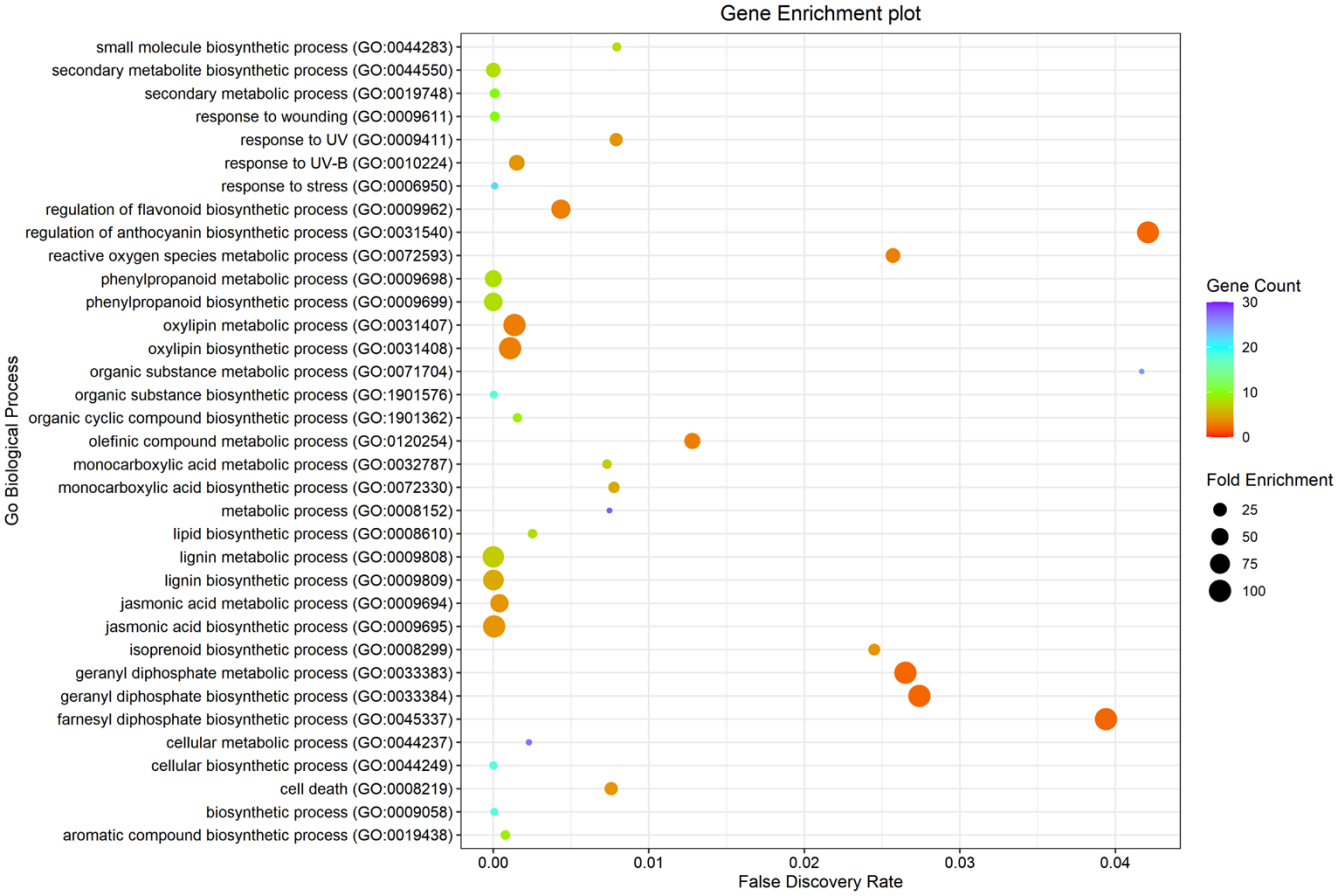
Gene Enrichment analysis using PANTHER (Mi et al., 2021) of significantly up-regulated genes upon GC treatment.

Gene ontology analysis of significantly upregulated genes in response to GC treatment from the expression analysis data was performed using PANTHER which revealed the involvement of these genes in major biological processes, mostly lignin biosynthesis, oxylipin biosynthesis, jasmonic acid biosynthesis and phenylpropanoid biosynthesis. A ggplot was plotted using R Studio (**Figure 9**).

### Phytochemical estimation of Piperine content in infiltrated leaves

**Figure 10:**
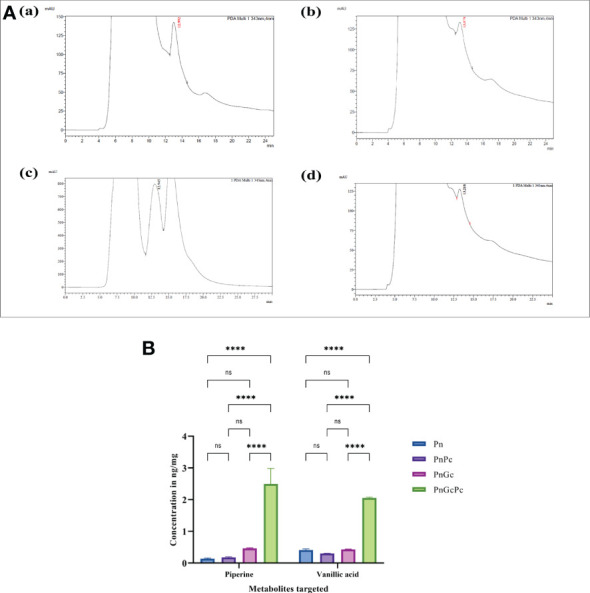
**(A)** HPLC Chromatograms of Piperine (343 nm, retention time 12.9 minutes) in detached *P. nigrum* leaves upon treatments. (a) Pn: Control *P. nigrum*, (b) PnPc: *P. capsici* infected *P. nigrum*, 24hpi, (c) PnGc: GC infiltrated *P. nigrum*, 24h, (d) PnGcPc: Gc infiltrated *P. nigrum*,24h, infected with *P. capsici*, 24hpi **(B)** Concentrations of Piperine and Vanillic acid (ng/mg) in *P. nigrum* detached leaves after different treatments as revealed by LC/MS-MS analysis (Pn: *P. nigrum* control, PnPc: *P. nigrum* infected with *P. capsici* (24hpi), PnGc: Glycol chitosan infiltrated *P. nigrum* (24h), PnGcPc: *P. nigrum* infected with *P. capsici* 24h post infiltration with Glycol chitosan, 24hpi) (Schmittgen and Livak, 2008). One-way ANOVA was performed to compare significance among treatments. Values are means of triplicates. Data are expressed as Mean ± SEM. p ≤ 0.0001 are represented by **** respectively and p ≥ 0.05 is represented by ns (not significant).

HPLC analysis of *P. nigrum* leaves after treatments revealed a higher amount of Piperine in GC-infiltrated leaves when compared to *P. capsici-infected*, and control leaves (**Figure 10A**). The concentration of Piperine in GC-infiltrated *P. nigrum* leaves was found to be 175 μg/mg of sample whereas in control *P. nigrum* and *P. capsici* infected *P. nigrum*, it was found to be 15.578 μg/mg sample and 9.85 μg/mg sample, respectively. The concentrations of Piperine in the samples were calculated by comparing it with the calibration curve of Piperine standard (Sigma Aldrich, USA) (**Supplementary Figure 1**). LC-MS/MS analysis of *P. nigrum* seedlings also showed a higher amount of piperine and vanillic acid in GC-treated seedlings (**Figure 10B**); other metabolites (Gallic acid, Chlorogenic acid, Picein, Catechin, Epicatechin, Caffeic acid, and Coumaric acid) were present below quantifiable levels. The respective chromatograms are given in **Supplementary Figure 2**.

### GC treatment significantly delayed disease incidence and progression in *Piper nigrum* plants

**Figure 11:**
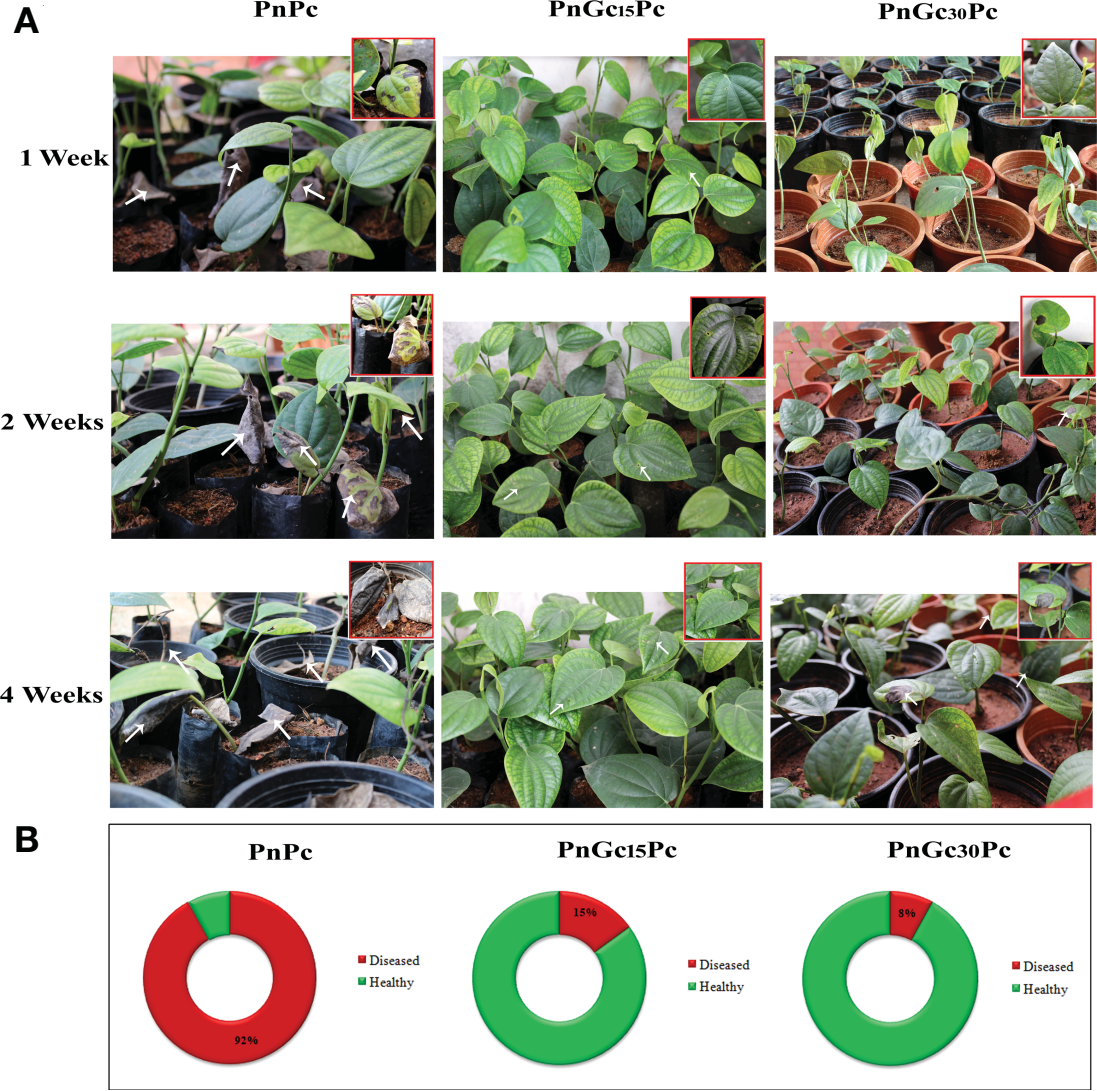
**(A)** Disease progression after 1, 2 and 4 weeks post infection with *P. capsici* in GC- infiltrated rooted cuttings of *P. nigrum*. PnPc: *P. nigrum* infected with *P. capsici*, PnGc_15_Pc: *P. nigrum* infected with *P. capsici*, 15 days post infiltration with GC, PnGc_30_Pc: *P. nigrum* infected with *P. capsici*, 30 days post infiltration with GC. The closer view of the plants that showed symptoms are given in the thumbnail and arrows indicate the site of lesion **(B)** Doughnut charts representing Disease Incidence in rooted cuttings of *P. nigrum*. Red indicates diseased and green indicates healthy plant percentage. PnPc: *P. nigrum* infected with *P. capsici*, PnGc_15_Pc: *P. nigrum* infected with *P. capsici*, 15 days post infiltration with GC, PnGc_30_Pc: *P. nigrum* infected with *P. capsici*, 30 days post infiltration with GC.

The infection progression after 1,2 and 4 weeks in rooted cuttings of *P. nigrum* after treatments is given in **Figure 11**. The first symptom of the infection in un-infiltrated leaves of GC-treated *P. nigrum* plants (15 days post infiltration and 30 days post infiltration) was seen at 7 days and 12 days post infection, respectively. In un-infiltrated leaves of the plant infected 15 days post infiltration with GC, none of the leaves showed infection progression whereas in un-infiltrated leaves of the plant infected 30 days post infiltration with GC, the lesion progressed on that particular leaf which eventually withered off, leaving the rest of the plant healthy. In infiltrated leaves (15- & 30-days post infiltration), the lesion was observed at 15 days post infection, which did not progress further. In the case of control *P. nigrum* plants, the lesion was seen after 48 hours of *P. capsici* infection, which progressed rapidly leading to the destruction of most of the plants within the first week of infection. The disease incidence in GC-treated plants (15 days post infiltration and 30 days post infiltration) and control *P. nigrum* plants was found to be 15%, 8% and 92%, respectively.

## Discussion

*Piper nigrum* (black pepper) is a tropical indigenous crop and its berries are the source of one of the world’s most widely and frequently used spices. However, ‘foot rot’ or ‘quick wilt’ disease caused by the oomycete *Phytophthora capsici*, is the major production constraint reported globally, wherever black pepper is cultivated. *Phytophthora capsici*, an oomycete (fungal-like) pathogen is one of the most devastating plant pathogens with severe economic repercussions on modern crops because of its ability to develop resistance against antifungal chemicals and bypass plant resistance genes (Larousse and Galiana, 2017). Chitosan as a plant protectant is generally recognized as safe (Raafat and Sahl, 2009) and was reportedly effective in protecting strawberries, tomatoes and grapes against *B. cinerea* (Muñoz and Moret, 2010) (Romanazzi et al., 2013). Different studies have shown that its effect on crop protection results from the induction of defense mechanisms (Sathiyabama et al., 2014) and direct antimicrobial activity (Goy et al., 2009).

Treatments with chitosan often require infiltration into the leaves to trigger a robust effect (Scalschi et al., 2015) which was confirmed by our observations in *Piper nigrum*. The efficiency of the infiltration method in stimulating host defense response in *P. nigrum* is suggestive of the potential of trunk injection for defense priming in black pepper plantations. *Piper nigrum* is a woody perennial climbing vine. Direct trunk injection (endotherapy) is reported as a promising way to deliver agrochemicals to many tree species while reducing environmental impacts and eliminating spray drift (Vandervoort, 2014).

*P. nigrum* is traditionally propagated by seeds and stem cuttings. However, seed production is inconsistent due to their short viability and high sterility (Atal and Banga, 1962), and hence the crop is largely propagated through cuttings for field plantations (Abbasi et al., 2010). In our study, pathogen infection assays and GC treatments were mostly performed in detached leaves of *P. nigrum*. Screening whole plants for disease scoring and elicitor treatment is cumbersome and hence detached leaf assay is established as the method of choice in *P. nigrum* under laboratory conditions (Duarte and Archer, 2003) (Mahadevan et al., 2016) (Paul et al., 2021). Moreover, detached leaf assay is a rapid, non-destructive method that is performed under a controlled environment and hence yields consistent results. The detached leaf assay has been generally used to evaluate natural products as potential fungicides (Abril et al., 2009) and for pathogen resistance screening (Miller-Butler et al., 2018). Experiments were also conducted in *P. nigrum* seedlings and cuttings to test the persistence of priming, its systemic effect on the plant and to validate the observations made in detached leaves on the potential impact of priming on the accumulation of Piperine.

GC infiltration significantly delayed the appearance of disease symptoms in detached leaves of mature plants and attached leaves of seedlings and cuttings. Pathogen DNA quantification clearly indicated a significant reduction of pathogen growth in leaves pre-treated with GC in detached leaves and seedlings. *In vitro* direct antifungal activity of oligo chitosan has been reported previously but we did not observe a direct inhibition of *P. capsici* under *in vitro* conditions (**Supplementary Figure 3**). An interesting observation that was made in our experiments on seedlings and cuttings was that the mechanical removal of infiltrated leaves after appearance of delayed visible symptoms helped in protecting the rest of the plant, as the infection did not spread further, unlike control seedlings which succumbed to the infection which spread to the whole plant in 5-6 days. It seems reasonable to infer that GC successfully stimulates systemic defense response in *P. nigrum*. It is known that in addition to local defense responses mediated through the production of ROS and NO, Chitosan triggers systemic defense in plants which is dependent on the phytohormones SA and ethylene as well as hormone-regulated expression of defense pathway genes and proteins as demonstrated in Tomato (Czékus et al., 2021).

It was consistently observed that foliar treatment of GC resulted in an ROS-dependent hypersensitive response in leaves of *P. nigrum*. This was evident by enhanced expression of NADPH oxidases, SOD and Peroxidase in GC-elicited leaves. Plasma membrane NADPH oxidases (NOXs), also named respiratory burst oxidase homologues (RBOHs), are critical generators of reactive oxygen species (ROS), which are signal molecules that regulate growth, development, and adaptation to various biotic and abiotic stresses in plants. NOXs-dependent ROS production is frequently induced by diverse phytohormones. The ROS commonly function downstream of, and interplay with hormone signallings, co-ordinately modulating plant development and stress tolerance (Sun et al., 2019).

In our observations, GC pre-treatment resulted in significant accumulation of Non-expressor of PR genes1 (*NPR1*) and Pathogenesis related protein1 (*PR1*) transcripts as indicated by qRT-PCR analysis of treated leaves. *NPR1* is known to be a master regulator of the salicylic acid (SA)-controlled signalling pathway. Activation of SA-mediated defense by pathogen attack activates SA defense in which antimicrobial proteins, such as the pathogenesis-related protein 1 (*PR1*), are produced in the secretory pathway for delivery into the apoplast (Wang et al., 2005). During SA defense, *NPR1* regulates the expression of *PR1* through interaction with TGACG motif-binding protein family (TGA) TFs (Fan and Dong, 2002).

Pathogenesis-related (PR) proteins are plant proteins that are induced in response to pathogen attacks and have been classified into 17 families (van Loon et al., 2006). In the present study, *PR1* and its acidic isoform *PR1a1, PR2* (β-1,3-glucanase), *PR5* (Thaumatin), *PR3* (Chitinase) and *PR12* (defensin) were some of the major PR gene transcripts that were majorly induced by pathogen infection/GC pre-treatment or both. It was interesting to note that their higher expression in GC-treated infected samples coincided with the inhibition of pathogen growth and enhanced host defense through the manifestation of HR. Overall the results indicate the potential of GC in triggering a defense reaction directed towards the accumulation of antimicrobial PR proteins for defending the pathogen attack. Previous transcriptomic studies have revealed that PR genes are significantly induced by both biotic and abiotic stresses, and this makes them one of the most promising candidates for developing multiple stress-tolerant crop varieties (Dai et al., 2016) (Ali et al., 2018).

The most abundant constituents of oomycete cell walls are glucans, polysaccharides that consist of linked glucose units. β-1,3 and β-1,6-glucan are the major components of oomycete cell walls, whereas cellulose, a β-1,4-glucan, forms a relatively small fraction (Aronson et al., 1967). Many plant species accumulate chitinases and ß-1,3-glucanases in response to infection by pathogens and treatments with the plant stress hormone, ethylene. It is noteworthy that GC infiltration stimulated significant expression of chitinase transcripts in *P. nigrum* leaves, which can be possibly attributed to chitosan treatment per se as chitosan is known to induce plant defense responses either directly or indirectly through a chitin elicitor receptor kinase 1 (CERK1) as reported in *Arabidopsis* by functioning as a Pathogen-Associated Molecular Pattern (PAMP) (Povero et al., 2011). However, it remains to be worked out whether *P. nigrum* possesses a CERK homologue that directly binds chitosan PAMPs. NON-RACE SPECIFIC DISEASE RESISTANCE-1(NDR1) is required for the full activation of PAMP-triggered immunity (PTI). It is hypothesized that the role of NDR1 in defense signalling is through the activation of the early events in both PTI and Effector-triggered immunity (ETI) (Knepper et al., 2011). Our preliminary observations in *P. nigrum* also suggest a critical role of *NDRI* gene in plant-pathogen interaction and host defense response.

Conforming to the augmented expression levels of critical genes of the defense signalling network, visible and direct manifestations of host plant defense were discernible by enhanced lignification and callose deposition of GC-infiltrated leaf tissue. The general phenylpropanoid pathway is associated with the shikimate route in plants. Phenylalanine ammonia-lyase (PAL) catalyzes the elimination of ammonia from Phe to produce *trans*-cinnamic acid (*t*-CA), which is subsequently converted to *p*-coumaric, caffeic, ferulic and sinapic acids. Increased upregulation of some of the genes involved in the phenylpropanoid pathway (**Supplementary Table 1**) was observed in response to foliar infiltration of GC. Phenylpropanoid metabolism and lignin biosynthetic pathways are known to be stimulated by stress and defense signalling. Our observations in *P. nigrum* showed an increase in lignin content in GC-treated leaves which coincided with an increase in RNA levels of key genes of the lignin biosynthesis pathway, which implicates a transcriptionally controlled lignin biosynthesis as a consequence of GC treatment. This conclusion is further supported by the phloroglucinol lignin staining assay. Glycol Chitosan-mediated priming also had a favourable effect on core phenylpropanoid pathway genes which include Phenylalanine ammonia lyase (*PAL), Cinnamate 4-Hydroxylase (C4H), CCR*, caffeoyl-CoA O-methyltransferase (*CCoAOMT*), Ferulate hydroxylase (*F5H*), which was significantly stimulated by GC. All these results strongly suggest a defensive role of GC by causing increased lignification. Specific pathways involved in GC-induced lignin synthesis and its structural specificity if any need to be ascertained. Studies in Poplar leaves exposed to Ozone stress demonstrated the contribution of structurally distinct stress-induced lignins in Poplar contributing to stress tolerance by causing a physical barrier or due to their antioxidant effect toward reactive oxygen species (Cabané et al., 2004). The higher accumulation of phenolics and lignin observed in the present study, corresponding to chitosan elicitation, are in agreement with previous reports (de Vega et al., 2021) (Nunes da Silva et al., 2021) and attest to the potential of chitosan elicitation in mounting host plant defense in *P. nigrum* against *P. capsici* infection.

Piperine, a cinnamoyl amide derivative is regarded as largely responsible for the pungent taste of black pepper. Despite its long history and worldwide use, the biosynthesis of Piperine and related amides has been enigmatic (Schnabel et al., 2020). In piperine, the aromatic part of the molecules is linked by an amide bond to a nitrogen-containing heterocycle, Piperidine. *P. nigrum* and other Piperaceae contain numerous combinations of aromatic and aliphatic amines linked to a diverse set of aromatic piperamides (Parmar et al., 1997) (Rios and Gómez-Calvario, 2019). The piperidine heterocycle is derived from L-lysine (Leistner and Spenser, 1973), whereas the aromatic part of piperine is likely to be derived from phenylpropanoid metabolism. In our study, the RNA levels of key genes in the Piperine biosynthetic pathway, especially the Cinnamoyl coA-mediated Phenylpropanoid route were preferentially enriched in response to GC which culminated in significant enhancement in Piperine content of detached mature leaves and seedlings. This is a significant finding because Piperine content is inherently low or negligible in *P. nigrum* leaves and the priming process evidently favours a marked increase in Piperine biosynthesis which led to a detectably enhanced level of Piperine content in leaves- an observation that has obvious commercial significance. Our results provide the first documentary evidence for the two-fold favourable effect of priming in plant protection and improvement of Piperine content.

Priming mechanisms are described at the epigenetic and the transcript or protein levels. In the priming phase, the plant remains in a sensitized state which enables the plant to exhibit an enhanced perception of stress and stronger defense upon subsequent pathogen attack (post-challenge priming stage). This phase is marked by the accumulation of Glucosinolates, Phytoalexins, Callose, Phenolics, Hormones such as SA and JA, Pathogenesis-related proteins and Histone modifications (Mauch-Mani et al., 2017). The hierarchical view on stress signalling, where metabolites are seen as final downstream products, has recently been complemented by findings that metabolites themselves function as stress signals (Schwachtje et al., 2019). However, the metabolic level as a mediator of priming has remained largely unexplored, even though large parts of metabolism are altered during stress (Bolton, 2009) (Krasensky and Jonak, 2012) (Fraire-Velazquez and Emmanuel, 2013). Many recent reports provide evidence that ‘metabolic imprints’ which result from persistent stress-induced changes in metabolite concentrations, metabolite ratios, or metabolic fluxes can prime responses to future environmental events. The kinetics of metabolic remodelling is largely dependent on the nature of the environmental stress, transcriptional activities, the architecture of the affected metabolic networks, and the set of transport rates and enzyme activities that act on the induced metabolite levels. Some induced metabolic responses may persist after the global metabolic state of the plant has recovered to the initial state due to delayed adjustment of a metabolite to the initial state, which causes a metabolite to be more abundant at the onset of a second stress event. Furthermore, some induced metabolic responses may be effectively permanent or even cumulative, e.g., due to the absence of a catabolic pathway or sequestration mechanism (Mackie et al., 2013). Currently, the knowledge of the short- to long-term dynamics of metabolic imprints is fragmented although it is a necessary component to assess the potential implication of metabolic remodelling associated with priming, especially in crops like *Piper nigrum* which is globally acclaimed because of its specialized secondary metabolites. Hence our future studies will determine the efficacy of defense priming in black pepper in terms of its persistence even after the elimination of the priming agent and its effect on the berries for its successful imprinting in successive generations.

## Conclusion

This paper reports for the first time the potential of defense priming as an efficient crop protection strategy in the woody perennial spice crop *Piper nigrum*, against infection by *Phytophthora capsici*. It was consistently observed that GC treatment offered protection from the severity of ‘quick wilt’ disease and caused a significant delay in the appearance of symptoms. Pre-treatment with GC of foliar samples resulted in induction of host innate immunity and HR as evidenced by significant accumulation of gene transcripts of ROS signalling, HR and phenylpropanoid biosynthesis and PR signalling pathways, in infiltrated leaves and seedlings which displayed a systemic spread of defense response. The priming effect was manifested through enhanced biosynthesis of lignin monomers, concomitant with induced expression of key genes of the lignin biosynthesis pathway, along with an enhanced accumulation of Piperine. The findings from this study establish a proof-of-concept of the potential of defense priming through stem/vine injection for protecting *P. nigrum* from *P. capsici* infection in nurseries and in plantations. Our results imply the potential application of glycol chitosan/defense priming as a promising adjunct to currently used quick wilt management practices. This data opens the way for in-depth mechanistic studies for identifying new potential natural elicitors that can prime the defense of *P. nigrum* sustainably.

## Data availability statement

The datasets presented in this study can be found in online repositories. The names of the repository/repositories and accession number(s) can be found in the article- BioProject accession number PRJNA318916.

## Author contributions

IM, MB and MS conceived and designed the experiments; IM and MB wrote the manuscript and performed the wet lab experiments. CM generated and analysed the transcriptome data of *Piper nigrum* and revised the manuscript. SK performed the genome mapping of transcriptome and interpreted the data. MS and RS led the project and revised the manuscript. MSK and BN helped in preparation of manuscript. All authors contributed to the article and approved the submitted version.

## References

[B1] Abbasi B. H. Ahmad N. Fazal H. Mahmood T. (2010). Conventional and modern propagation techniques in piper nigrum. J. Med. Plants Res. 4 (1), 7–12. doi: 10.5897/JMPR09.025

[B2] Abril M. Curry K. J. Smith B. J. Delucca A. J. Boue S. Wedge D. E. (2009). Greenhouse and field evaluation of the natural saponin CAY-1 for control of several strawberry diseases. Int. J. Fruit Sci. 9 (3), 211–220. doi: 10.1080/15538360903241153

[B3] Ahmad S. Gordon-Weeks R. Pickett J. Ton J. (2010). Natural variation in priming of basal resistance: From evolutionary origin to agricultural exploitation. Mol. Plant Pathol. 11, 817–827. doi: 10.1111/j.1364-3703.2010.00645.x 21029325 PMC6640509

[B4] Ali S. Ganai B. A. Kamili A. N. Bhat A. A. Mir Z. A. Bhat J. A.. (2018). Pathogenesis-related proteins and peptides as promising tools for engineering plants with multiple stress tolerance. Microbiol. Res. Elsevier GmbH; 212–213, 29–37. doi: 10.1016/j.micres.2018.04.008 29853166

[B5] Anandaraj M. Sarma Y. R. (1995). Diseases of black pepper (Piper nigrum l.) and their management. J. Spices Aromatic Crops 4 (1), 17–23. Available at: https://updatepublishing.com/journal/index.php/josac/article/view/4343.

[B6] Aronson J. M. Cooper B. A. Fuller M. S. (1967). Glucans of oomycete cell walls. New Ser. 155, 332–335. doi: 10.1126/science.155.3760.332 17792061

[B7] Atal C. Banga S. (1962). Phytochemical studies on stem of p. longum. Indian Jour Pharm. 24, 105.

[B8] Beneloujaephajri E. Costa A. L’Haridon F. Métraux J. P. Binda M. (2013). Production of reactive oxygen species and wound-induced resistance in arabidopsis thaliana against botrytis cinerea are preceded and depend on a burst of calcium. BMC Plant Biol. 13 (1), 160. doi: 10.1186/1471-2229-13-160 24134148 PMC4016300

[B9] Bolton M. D. (2009). Primary metabolism and plant defense-fuel for the fire. MPMI 22 (5), 487–497. doi: 10.1094/MPMI-22-5-0487 19348567

[B10] Boratyn G. M. Thierry-Mieg J. Thierry-Mieg D. Busby B. Madden T. L. (2019). Magic-BLAST, an accurate RNA-seq aligner for long and short reads. BMC Bioinf. 20 (1), 405. doi: 10.1186/s12859-019-2996-x PMC6659269 31345161

[B11] Cabané M. Pireaux J. C. Léger E. Weber E. Dizengremel P. Pollet B.. (2004). Condensed lignins are synthesized in poplar leaves exposed to ozone. Plant Physiol. 134 (2), 586–594. doi: 10.1104/pp.103.031765 14730080 PMC344535

[B12] Chassot C. Buchala A. Schoonbeek H. J. Métraux J. P. Lamotte O. (2008). Wounding of arabidopsis leaves causes a powerful but transient protection against botrytis infection. Plant J. 55 (4), 555–567. doi: 10.1111/j.1365-313X.2008.03540.x 18452590

[B13] Chung C. L. Longfellow J. M. Walsh E. K. Kerdieh Z. van Esbroeck G. Balint-Kurti P.. (2010). Resistance loci affecting distinct stages of fungal pathogenesis: Use of introgression lines for QTL mapping and characterization in the maize - setosphaeria turcica pathosystem. BMC Plant Biol. 10, 103. doi: 10.1186/1471-2229-10-103 20529319 PMC3017769

[B14] Czékus Z. Iqbal N. Pollák B. Martics A. Ördög A. Poór P. (2021). Role of ethylene and light in chitosan-induced local and systemic defence responses of tomato plants. J. Plant Physiol. 263, 153461. doi: 10.1016/j.jplph.2021.153461 34217837

[B15] Dai L. Wang D. Xie X. Zhang C. Wang X. Xu Y.. (2016). The novel gene VpPR4-1 from vitis pseudoreticulata increases powdery mildew resistance in transgenic vitis vinifera l. Front. Plant Sci. 7 (MAY2016). doi: 10.3389/fpls.2016.00695 PMC4882328 27303413

[B16] de Vega D. Holden N. Hedley P. E. Morris J. Luna E. Newton A. (2021). Chitosan primes plant defence mechanisms against botrytis cinerea, including expression of Avr9/Cf-9 rapidly elicited genes. Plant Cell Environ. 44 (1), 290–303. doi: 10.1111/pce.13921 33094513 PMC7821246

[B17] Duarte M. Archer S. A. (2003). *In vitro* toxin production by fusarium solani f. sp. piperis. Fitopatol. Bras. 28 (3), 229–235. doi: 10.1590/S0100-41582003000300002

[B18] Fan W. Dong X. (2002). *In vivo* interaction between NPR1 and transcription factor TGA2 leads to salicylic acid-mediated gene activation in arabidopsis. Plant Cell. 14 (6), 1377–89. doi: 10.1105/tpc.001628 PMC150786 12084833

[B19] Foster J. M. Hausbeck M. K. (2010). Managing phytophthora crown and root rot in bell pepper using fungicides and host resistance. Plant Dis. 94 (6), 697–702. doi: 10.1094/PDIS-94-6-0697 30754316

[B20] Fraire-Velazquez S. Emmanuel V. (2013). “Abiotic stress in plants and metabolic responses,” in Abiotic stress - plant responses and applications in agriculture (London: InTech). doi: 10.5772/54859

[B21] Fukushima R. S. Kerley M. S. (2011). Use of lignin extracted from different plant sources as standards in the spectrophotometric acetyl bromide lignin method. J. Agric. Food Chem. 59 (8), 3505–3509. doi: 10.1021/jf104826n 21375240

[B22] Geetha R. G. Chandrika S. K. N. Saraswathy G. G. Sivakumari A. N. Sakuntala M. (2021). Ros dependent antifungal and anticancer modulations of piper colubrinum osmotin. Molecules 26 (8), 2239. doi: 10.3390/molecules26082239 33924432 PMC8070354

[B23] Goy R. C. de Britto D. Assis O. B. G. (2009). A review of the antimicrobial activity of chitosan. Polímeros 19 (3), 241–247. doi: 10.1590/S0104-14282009000300013

[B24] Hao X. Li L. Hu Y. Zhou C. Wang X. Wang L.. (2016). Transcriptomic analysis of the effects of three different light treatments on the biosynthesis of characteristic compounds in the tea plant by RNA-seq. Tree Genet. Genomes 12 (6), 118. doi: 10.1007/s11295-016-1071-2

[B25] Hausbeck M. K. Lamour K. H. (2004). Phytophthora capsici on vegetable crops: Research progress and management challenges. Plant Dis. 88 (12), 1292–1303. doi: 10.1094/PDIS.2004.88.12.1292 30795189

[B26] Hu L. Xu Z. Wang M. Fan R. Yuan D. Wu B.. (2019). The chromosome-scale reference genome of black pepper provides insight into piperine biosynthesis. Nat. Commun. 10 (1), 4702. doi: 10.1038/s41467-019-12607-6 31619678 PMC6795880

[B27] Jin L. Mackey D. M. (2017). “Measuring callose deposition, an indicator of cell wall reinforcement, during bacterial infection in arabidopsis,” in Methods in molecular biology (Clifton, N.J.: Humana Press Inc) 1578. 195–205. doi: 10.1007/978-1-4939-6859-6_16 28220426

[B28] Juárez S. P. D. Mangano S. Estevez J. M. (2015). Improved ROS measurement in root hair cells. Methods Mol. Biol. 1242, 67–71. doi: 10.1007/978-1-4939-1902-4_6 25408444

[B29] Knepper C. Savory E. A. Day B. (2011). Arabidopsis NDR1 is an integrin-like protein with a role in fluid loss and plasma membrane-cell wall adhesion. Plant Physiol. 156 (1), 286–300. doi: 10.1104/pp.110.169656 21398259 PMC3091050

[B30] Krasensky J. Jonak C. (2012). Drought, salt, and temperature stress-induced metabolic rearrangements and regulatory networks. J. Exp. Bot. 63, 1593–1608. doi: 10.1093/jxb/err460 22291134 PMC4359903

[B31] Krishnamoorthy B. Parthasarathy V. A. (2009). Improvement of black pepper. CAB Rev.: Perspect. Agricul. Vet. Sci. Nutr. Natural Resour. 4 (085), 1749–8848. doi: 10.1079/PAVSNNR20094085

[B32] Krishnan A. Mahadevan C. Mani T. Sakuntala M. (2015). Virus-induced gene silencing (VIGS) for elucidation of pathogen defense role of serine/threonine protein kinase in the non-model plant piper colubrinum link. Plant Cell Tissue Organ Cult. 122 (2), 269–283. doi: 10.1007/s11240-015-0764-9

[B33] Krzywinski M. Schein J. Birol I. Connors J. Gascoyne R. Horsman D.. (2009). Circos: An information aesthetic for comparative genomics. Genome Res. 19 (9), 1639–1645. doi: 10.1101/gr.092759.109 19541911 PMC2752132

[B34] Lamour K. Hausbeck M. (2000). Mefenoxam insensitivity and the sexual stage of phytophthora capsici in Michigan cucurbit fields. Phytopathology 90 (4), 396–400. doi: 10.1094/PHYTO.2000.90.4.396 18944590

[B35] Larousse M. Galiana E. (2017). Microbial partnerships of pathogenic oomycetes. PloS Pathog. 13 (1), e1006028. doi: 10.1371/journal.ppat.1006028 28125714 PMC5268404

[B36] Leistner E. Spenser I. D. (1973). Biosynthesis of the piperidine nucleus. incorporation of chirally labeled cadaverine-1-3H. J. Am. Chem. Soc 95 (14), 4715–4725. doi: 10.1021/ja00795a041 4199817

[B37] Lopez-Moya F. Escudero N. Zavala-Gonzalez E. A. Esteve-Bruna D. Blázquez M. A. Alabadí D.. (2017). Induction of auxin biosynthesis and WOX5 repression mediate changes in root development in arabidopsis exposed to chitosan. Sci. Rep. 7 (1), 16813. doi: 10.1038/s41598-017-16874-5 PMC5711845 29196703

[B38] Mackie A. Keseler I. M. Nolan L. Karp P. D. Paulsen I. T. (2013). Dead end metabolites - defining the known unknowns of the e. coli metabolic network. PloS One 8 (9), e75210. doi: 10.1371/journal.pone.0075210 24086468 PMC3781023

[B39] Mahadevan C. Krishnan A. Saraswathy G. G. Surendran A. Jaleel A. Sakuntala M. (2016). Transcriptome- assisted label-free quantitative proteomics analysis reveals novel insights into piper nigrum–phytophthora capsici phytopathosystem. Front. Plant Sci. 7 (JUNE2016). doi: 10.3389/fpls.2016.00785 PMC4913111 27379110

[B40] Mani T. Manjula S. (2020). Cloning and analysis of promoter elements of a Ser/Thr protein kinase gene homologue from piper colubrinum link. Indian J. Biotechnol. 19, 9–16. Available at: http://nopr.niscpr.res.in/handle/123456789/55168.

[B41] Mani T. Sivakumar K. C. Manjula S. (2012). Expression and functional analysis of two osmotin (PR5) isoforms with differential antifungal activity from piper colubrinum: Prediction of structure-function relationship by bioinformatics approach. Mol. Biotechnol. 52 (3), 251–261. doi: 10.1007/s12033-011-9489-0 22207456

[B42] Mauch-Mani B. Baccelli I. Luna E. Flors V. (2017). Defense priming: An adaptive part of induced resistance. Annu. Rev. Plant Biol. 68, 485–512. doi: 10.1146/annurev-arplant-042916-041132 28226238

[B43] Mi H. Ebert D. Muruganujan A. Mills C. Albou L. P. Mushayamaha T.. (2021). PANTHER version 16: A revised family classification, tree-based classification tool, enhancer regions and extensive API. Nucleic Acids Res. 49 (D1), D394–D403. doi: 10.1093/nar/gkaa1106 33290554 PMC7778891

[B44] Miller-Butler M. A. Smith B. J. Babiker E. M. Kreiser B. R. Blythe E. K. (2018). Comparison of whole plant and detached leaf screening techniques for identifying anthracnose resistance in strawberry plants. Plant Dis. 102 (11), 2112–2119. doi: 10.1094/PDIS-08-17-1138-RE 30211658

[B45] Moreira-Vilar F. C. Siqueira-Soares R. D. C. Finger-Teixeira A. de Oliveira D. M. Ferro A. P. da Rocha G. J.. (2014). The acetyl bromide method is faster, simpler and presents best recovery of lignin in different herbaceous tissues than klason and thioglycolic acid methods. PloS One 9 (10), e110000. doi: 10.1371/journal.pone.0110000 25330077 PMC4212577

[B46] Muñoz Z. Moret A. (2010). Sensitivity of botrytis cinerea to chitosan and acibenzolar-s-methyl. Pest Manag. Sci. 66 (9), 974–979. doi: 10.1002/ps.1969 20730989

[B47] Negi A. George Kokkat J. Jasrotia R. S. Madhavan S. Jaiswal S. Angadi U. B.. (2021). Drought responsiveness in black pepper (Piper nigrum l.): Genes associated and development of a web-genomic resource. Physiol. Plant 172 (2), 669–683. doi: 10.1111/ppl.13308 33305409

[B48] Negi A. Singh K. Jaiswal S. Kokkat J. G. Angadi U. B. Iquebal M. A.. (2022). Rapid genome-wide location-specific polymorphic SSR marker discovery in black pepper by GBS approach. Front. Plant Sci. 13. doi: 10.3389/fpls.2022.846937 PMC9197322 35712605

[B49] Nunes da Silva M. Santos C. S. Cruz A. López-Villamor A. Vasconcelos M. W. (2021). Chitosan increases pinus pinaster tolerance to the pinewood nematode (Bursaphelenchus xylophilus) by promoting plant antioxidative metabolism. Sci. Rep. 11 (1), 3781. doi: 10.1038/s41598-021-83445-0 33580134 PMC7881030

[B50] Özyilmaz Ü Benlioglu K. (2013). Enhanced biological control of phytophthora blight of pepper by biosurfactant-producing pseudomonas. Plant Pathol. J. 29 (4), 418–426. doi: 10.5423/PPJ.OA.11.2012.0176 25288970 PMC4174822

[B51] Parmar V. S. Jain S. C. Bisht K. S. Jain R. Taneja P. Jha A.. (1997). Phytochemistry of the genus piper. Phytochemistry 46 (4), 597–673. doi: 10.1016/S0031-9422(97)00328-2

[B52] Paul T. Nysanth N. S. Yashaswini M. S. Anith K. N. (2021). Inoculation with bacterial endophytes and the fungal root endophyte, piriformospora indica improves plant growth and reduces foliar infection by phytophthora capsici in black pepper. J. Trop. Agric. 59 (2), 224–235.

[B53] Povero G. Loreti E. Pucciariello C. Santaniello A. di Tommaso D. di Tommaso G.. (2011). Transcript profiling of chitosan-treated arabidopsis seedlings. J. Plant Res. 124 (5), 619–629. doi: 10.1007/s10265-010-0399-1 21240536

[B54] Pradhan Mitra P. Loqué D. (2014). Histochemical staining of arabidopsis thaliana secondary cell wall elements. J. Visualized Experiments 87, 51381. doi: 10.3791/51381 PMC4186213 24894795

[B55] Pusztahelyi T. (2018). Chitin and chitin-related compounds in plant–fungal interactions. Mycology 9, 189–201. doi: 10.1080/21501203.2018.1473299 30181925 PMC6115883

[B56] Qi M. Yang Y. (2002). Quantification of magnaporthe grisea during infection of rice plants using real-time polymerase chain reaction and northern Blot/Phosphoimaging analyses. Phytopathology 92 (8), 870–876. doi: 10.1094/PHYTO.2002.92.8.870 18942966

[B57] Raafat D. Sahl H. G. (2009). Chitosan and its antimicrobial potential - a critical literature survey. Microbial. Biotechnol. 2, 186–201. doi: 10.1111/j.1751-7915.2008.00080.x PMC3815839 21261913

[B58] Rios M. Y. Gómez-Calvario V. (2019). 1H and 13C NMR data, occurrence, biosynthesis, and biological activity of piper amides. Magnetic Resonance Chem. 57, 993. doi: 10.1002/mrc.4941 30779382

[B59] Romanazzi G. Feliziani E. Santini M. Landi L. (2013). Effectiveness of postharvest treatment with chitosan and other resistance inducers in the control of storage decay of strawberry. Postharvest Biol. Technol. 75, 24–27. doi: 10.1016/j.postharvbio.2012.07.007

[B60] Sathiyabama M. Akila G. Einstein Charles R. (2014). Chitosan-induced defence responses in tomato plants against early blight disease caused by alternaria solani (Ellis and martin) sorauer. Arch. Phytopathol. Plant Prot. 47 (14), 1777–1787. doi: 10.1080/03235408.2013.858423

[B61] Satyagopal K. Sushil S. N. Jeyakumar P. Shankar G. Sharma O. P. (2014). AESA based IPM package for black pepper National Institute of Plant Health Management, Hyderabad: Balaji Scans Pvt. Ltd. Press. Page 38.

[B62] Scalschi L. Sanmartín M. Camañes G. Troncho P. Sánchez-Serrano J. J. García-Agustín P.. (2015). Silencing of OPR3 in tomato reveals the role of OPDA in callose deposition during the activation of defense responses against botrytis cinerea. Plant J. 81 (2), 304–315. doi: 10.1111/tpj.12728 25407262

[B63] Schmittgen T. D. Livak K. J. (2008). Analyzing real-time PCR data by the comparative CT method. Nat. Protoc. 3 (6), 1101–1108. doi: 10.1038/nprot.2008.73 18546601

[B64] Schnabel A. Cotinguiba F. Athmer B. Yang C. Westermann B. Schaks A.. (2020). A piperic acid CoA ligase produces a putative precursor of piperine, the pungent principle from black pepper fruits. Plant J. 102 (3), 569–581. doi: 10.1111/tpj.14652 31837062

[B65] Schwachtje J. Whitcomb S. J. Firmino A. A. P. Zuther E. Hincha D. K. Kopka J. (2019). Induced, imprinted, and primed responses to changing environments: Does metabolism store and process information? Front. Plant Sci. 10. doi: 10.3389/fpls.2019.00106 PMC6381073 30815006

[B66] Singh P. Plant defense priming: A new tool for sustainable global food security. (Rajasthan,India: Agrobios Research) (2021) Pages 133–153

[B67] Sun L. R. Zhao Z. J. Hao F. S. (2019). NADPH oxidases, essential players of hormone signalings in plant development and response to stresses. Plant Signaling Behav. 14 (11), 1657343. doi: 10.1080/15592324.2019.1657343 PMC6804714 31431139

[B68] Uge E. Sulandari S. Hartono S. Somowiyarjo S. (2018). The effect of chitosan application against plant growth and intensity of stunting disease on black pepper (Piper nigrum l.) seedlings. Jurnal Perlindungan Tanaman Indonesia 22 (2), 224. doi: 10.22146/jpti.25453

[B69] Upadhyay V. Sharma N. Joshi H. M. Malik A. Mishra M. Singh B. P.. (2013). Development and validation of rapid RP-HPLC method for estimation of piperine in piper nigrum l. Int. J. Herbal Med. 1 (4), 6–9.

[B70] Vandervoort C. (2014). Trunk injection: A discriminating delivering system for horticulture crop IPM. Entomol. Ornithol. Herpetol. 03 (02), 1–7. doi: 10.4172/2161-0983.1000126

[B71] van Hulten M. Pelser M. van Loon L. C. Pieterse C. M. J. Ton J. (2006). Costs and benefits of priming for defense in arabidopsis. PNAS 103 (14), 5602–5607. doi: 10.1073/pnas.0510213103 16565218 PMC1459400

[B72] van Loon L. C. Rep M. Pieterse C. M. J. (2006). Significance of inducible defense-related proteins in infected plants. Annu. Rev. Phytopathol. 44, 135–162. doi: 10.1146/annurev.phyto.44.070505.143425 16602946

[B73] Wang D. Weaver N. D. Kesarwani M. Dong X. (2005). Induction of protein secretory pathway is required for systemic acquired resistance. Sci. New Ser. 308, 1036-40. doi: 10.1126/science.1108791 15890886

[B74] Westman S. M. Kloth K. J. Hanson J. Ohlsson A. B. Albrectsen B. R. (2019). Defence priming in arabidopsis – a meta-analysis. Sci. Rep. 9 (1), 13309. doi: 10.1038/s41598-019-49811-9 31527672 PMC6746867

[B75] Zuluaga A. P. Vega-Arreguín J. C. Fei Z. Ponnala L. Lee S. J. Matas A. J.. (2016). Transcriptional dynamics of phytophthora infestans during sequential stages of hemibiotrophic infection of tomato. Mol. Plant Pathol. 17 (1), 29–41. doi: 10.1111/mpp.12263 25845484 PMC6638332

