# Supplementary material for: ‘Priming’ protects Piper nigrum L. from Phytophthora capsici through reinforcement of phenylpropanoid pathway and possible enhancement of Piperine biosynthesis
Source: Front Plant Sci. 2022 Dec 6;13:1072394. doi: 10.3389/fpls.2022.1072394 (PMC11908380; doi:10.3389/fpls.2022.1072394)

**Supplementary Figure 1:** Calibration curve of Piperine standard (Sigma Aldrich, USA) with R^2^=0.9937. x-axis represents the area under the peak and y-axis represents the different concentrations in ppm.


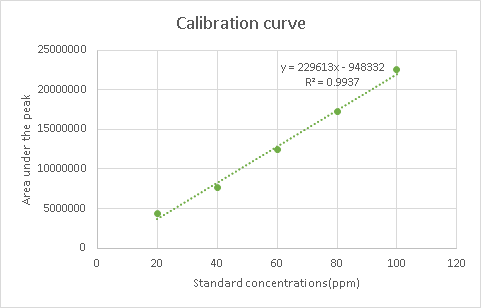


**Supplementary Figure 2:** The metabolites targeted in *P. nigrum* seedling samples are given in the figure below followed by LC-MS/MS chromatograms of the same: (a) *P. nigrum*, (b) *P. nigrum* infected with *P. capsici*, 24hpi, (c) *P. nigrum* infiltrated with GC, 24h and (d) *P. nigrum* infected with *P. capsici*, 24h post infiltration with GC, 24hpi.

| **Metabolites Targeted** | |
| --- | --- |
| **Gallic acid** | **Coumaric acid** |
| **Chlorogenic acid** | **Ferulic acid** |
| **Picein** | **Taxifolin** |
| **Catechin** | **Myricetin** |
| **Epicatechin** | **Coumarin** |
| **Caffeic acid** | **Quercetin** |
| **Vanillic acid** | **Narigenin** |
| **Kaempferol** | **Piperine** |


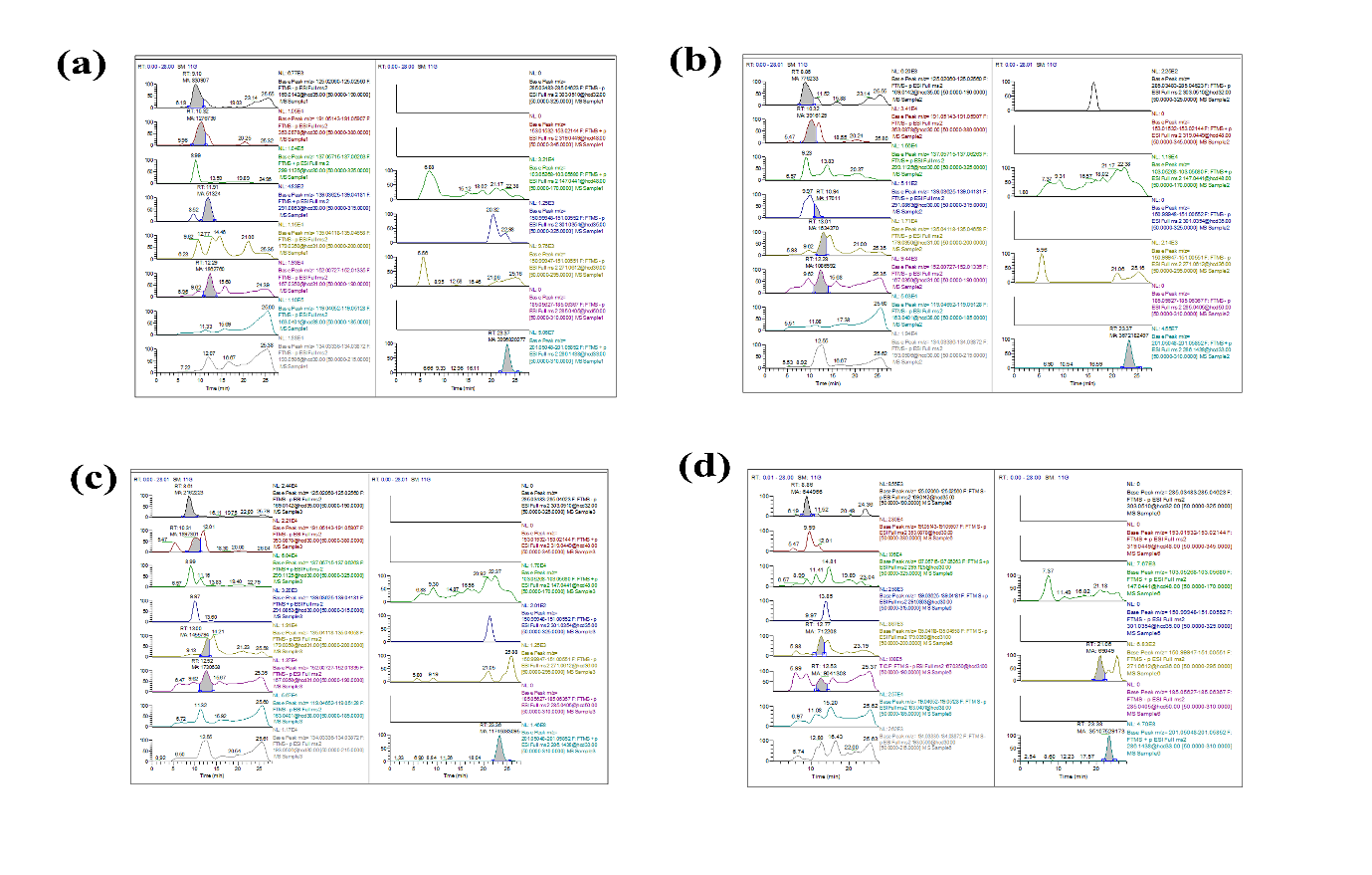


**Supplementary Figure 3:** Sytox green Dead cell staining was used to visualize the direct inhibitory effect of glycol chitosan (1mg/ml) on *P.capsici* using confocal microscopy under 20X magnification (Scale bar: 20μm). Green fluorescence indicates dead hyphae. It was observed that chitosan does not cause hyphal death unlike the positive control which contained heat- killed hyphae.


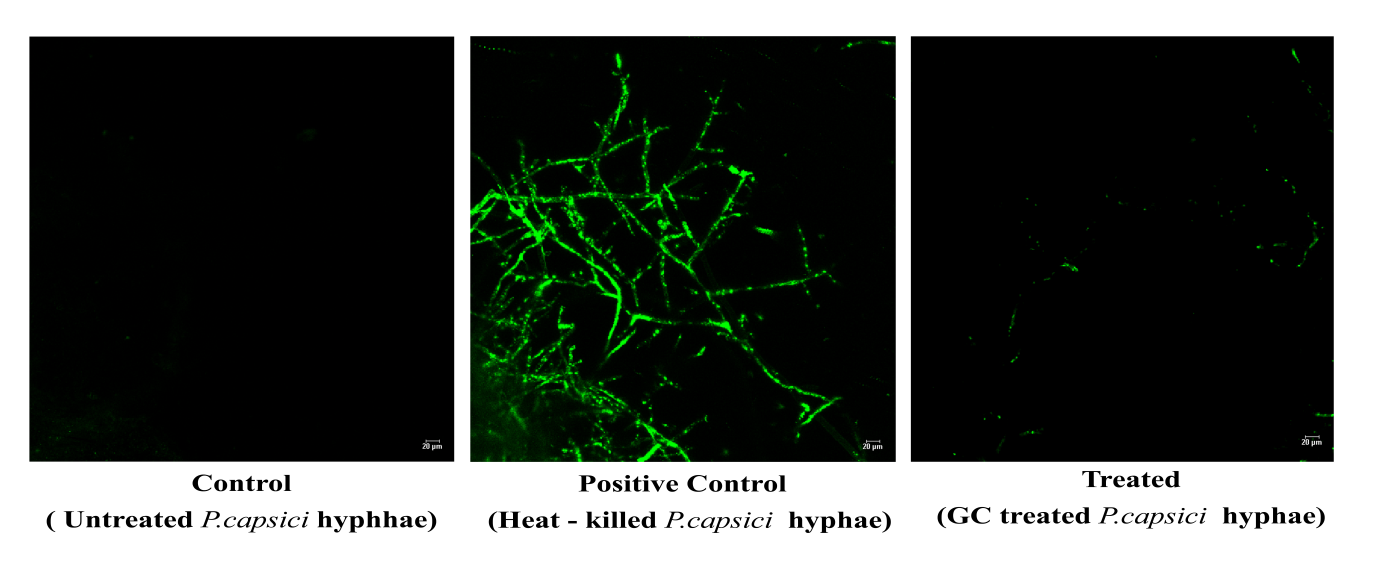

Supplement: Supplementary file 1 [file DataSheet_1.docx]
